# Sulfur‐Functionalized MOF via Ligand Additive‐Stabilized SALE for Efficient Hg^2+^ Ion Removal

**DOI:** 10.1002/smll.202503637

**Published:** 2025-07-09

**Authors:** Cheongwon Bae, Ho‐Jun Cho, Ju Hyun Kim, Xiaohui Song, Juyeong Kim

**Affiliations:** ^1^ Department of Chemistry Gyeongsang National University Jinju 52828 South Korea; ^2^ Research Institute of Advanced Chemistry Gyeongsang National University Jinju 52828 South Korea; ^3^ Department of Chemistry Dongguk University Seoul 04620 South Korea; ^4^ School of Materials Science and Engineering Hefei University of Technology Anhui Province 230009 China; ^5^ Engineering Research Center of High Performance Copper Alloy Materials and Processing Ministry of Education Hefei University of Technology Hefei 230009 China

**Keywords:** ligand additive stabilization, mercury ion removal, metal–organic framework, solvent‐assisted linker exchange, sulfur functionalization

## Abstract

Metal–organic frameworks (MOFs) are versatile materials used in adsorption and separation. Post‐synthetic modification, especially solvent‐assisted linker exchange (SALE), enables precise ligand functionalization while maintaining framework integrity. However, incorporating linkers with Lewis basic sites presents challenges due to their strong coordination tendencies, which can disrupt framework stability. In this work, a ligand additive‐stabilized SALE is introduced to incorporate 2‐mercaptoimidazole into zeolitic imidazolate framework‐8 (ZIF‐8), preserving its crystallinity and porosity while enabling controlled functionalization. A systematic study is conducted to correlate SALE parameters—including exchange ligand concentration, reaction time, and solvent environment—with morphological changes and ligand incorporation efficiency. Quantitative image analysis of MOF particle curvature revealed the relationship between ligand exchange dynamics and framework stability, providing insights into SALE‐induced structural transformations. The sulfur‐functionalized ZIF‐8 exhibits a high Hg^2+^ adsorption capacity of 1504 mg g^−1^ and excellent recyclability. It showed Hg^2+^ removal efficiency (>95%) across a wide pH range and retained 95% of its initial capacity after cycles. In a multi‐ion system containing additional metal ions, the material demonstrated ≈100% removal efficiency for Hg^2+^, confirming its practical applicability in complex environments. This ligand additive‐stabilized SALE provides a reliable approach for introducing sulfur functionalities into MOFs, opening opportunities for enhanced environmental remediation.

## Graphical abstract

A ligand additive‐stabilized SALE method enables the incorporation of 2‐mercaptoimidazole into ZIF‐8 while maintaining its structure and porosity. Systematic analysis reveals how SALE conditions affect morphology and ligand exchange. The sulfur‐functionalized ZIF‐8 achieves high Hg^2+^ adsorption capacity and selectivity, retaining performance after multiple cycles. This approach offers a stable, controllable route for MOF functionalization, advancing applications in environmental remediation.

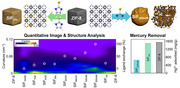

## 1 Introduction

Metal–organic frameworks (MOFs) are crystalline materials composed of metal ions coordinated with organic ligands, forming porous structures.^[^ ^1^, ^2^, ^3^ ^]^ Their tunable crystal structures, pore sizes, and chemical environments make MOFs highly adaptable for various applications, including adsorption,^[^ ^4^, ^5^, ^6^ ^]^ separation,^[^ ^7^, ^8^, ^9^ ^]^ catalysis,^[^ ^10^, ^11^, ^12^ ^]^ drug delivery,^[^ ^13^, ^14^, ^15^ ^]^ and electronics.^[^ ^16^, ^17^, ^18^, ^19^ ^]^ This versatility renders MOFs more advantageous than traditional porous materials like silica, zeolites, and activated carbon.

Reticular chemistry is grounded in the idea that crystalline porous materials can be assembled through the deliberate linkage of rigid secondary building units (SBUs) and organic linkers with well‐defined geometries.^[^ ^20^, ^21^, ^22^ ^]^ This strategy has led to a set of preferred topologies that can be reliably targeted and reproduced. The use of SBUs enabled the construction of permanently porous frameworks and, through the isoreticular principle, allowed systematic expansion of MOF structures by varying linker size and functionality without altering the underlying topology. This has made it possible to achieve ultrahigh porosity and tunable pore apertures. These advances demonstrate that MOFs are not products of empirical assembly but rather of deliberate, modular chemical design guided by geometric and topological principles. However, experimental realization does not always reflect the intended design: deviations in reaction kinetics or thermodynamics may lead to alternate framework topologies, framework interpenetration, or even the complete failure to form the desired MOF for certain node‐linker combinations.^[^ ^23^, ^24^ ^]^

To address these challenges, post‐synthetic modification (PSM) techniques have been developed, enabling targeted modifications of existing MOFs by introducing functional groups,^[^ ^25^, ^26^, ^27^ ^]^ replacing metal centers,^[^ ^28^, ^29^, ^30^ ^]^ or exchanging organic linkers.^[^ ^31^, ^32^, ^33^, ^34^ ^]^ PSM offers a more efficient approach to obtaining application‐specific MOFs by reducing the need for extensive synthetic optimization, thereby saving time and cost. In particular, the chemical properties of MOF porous structures can be significantly influenced by the nature of the organic linkers.^[^ ^35^, ^36^ ^]^ Solvent‐assisted linker exchange (SALE) has proven to be an effective method for modifying MOFs while maintaining their structural and morphological stability.^[^ ^37^, ^38^, ^39^ ^]^ Unlike direct introduction of functional groups, which can expose the MOF to undesirable chemical reactions and structural deformation, SALE in compatible solvent environments preserves the integrity of the framework while achieving the desired chemical modifications.

SALE has been commonly applied to MOFs containing multidentate organic linkers, such as 1,4‐benzenedicarboxylic acid and 1,3,5‐benzenetricarboxylic acid, where all Lewis basic sites are coordinated with metal ions within the framework.^[^ ^40^, ^41^, ^42^, ^43^ ^]^ The introduction of exchange linkers containing additional Lewis basic elements, such as ZIF8‐A and UiO‐68‐NHC, is of significant interest due to their potential to create additional reactive sites within the MOF pore for gas adsorption and catalysis. For instance, amine‐functionalized ZIF‐8 (ZIF8‐A) was synthesized through SALE by exchanging 2‐methylimidazole with 3‐amino‐1,2,4‐triazole.^[^ ^44^ ^]^ This modification significantly enhanced CO_2_/N_2_ and CO_2_/CH_4_ selectivity. In addition, the Zr‐based MOF functionalized with N‐heterocyclic carbene (UiO‐68‐NHC), synthesized via SALE, enabled the quantitative conversion of CO_2_ into methanol through silane‐mediated reduction.^[^ ^45^ ^]^ This process exhibited high catalytic activity and selectivity, overcoming the limitations of previous homogeneous NHC catalysts. However, incorporating such exchange linkers can be challenging, as the additional Lewis basic sites may participate in unwanted coordination with the metal center or undergo side reactions with nucleophilic sites within the framework during the SALE process, often leading to morphological and structural deformation of the MOF.

We developed ligand additive‐stabilized SALE to incorporate the additional Lewis base‐containing ligand, 2‐mercaptoimidazole, into zeolitic imidazolate framework‐8 (ZIF‐8), successfully maintaining its original structure and morphology. 2‐Mercaptoimidazole is an organic linker containing a terminal sulfur group and nitrogen sites capable of coordinating with metals. Its structure, with three Lewis basic sites, presents challenges for integration into the ZIF‐8 framework using conventional synthetic methods. While previous studies have explored the incorporation of sulfur‐containing ligands into ZIF‐8 via SALE, such modifications often resulted in structural deformation of the framework.^[^ ^46^, ^47^, ^48^ ^]^

In this study, we found that introducing 2‐methylimidazole as an additive during the SALE process with 2‐mercaptoimidazole significantly enhanced the structural stability of ZIF‐8. Incorporating sulfur‐containing ligands not only facilitates physical interactions with guest molecules based on pore size and structure but also imparts additional chemical functionality to the framework.^[^ ^49^, ^50^, ^51^ ^]^ Notably, sulfur exhibits strong interactions with heavy metal ions,^[^ ^52^, ^53^, ^54^, ^55^ ^]^ noble metals,^[^ ^56^, ^57^ ^]^ and specific gases such as CO_2_,^[^ ^58^, ^59^ ^]^ thereby improving molecular adsorption‐desorption selectivity and separation performance, making it a promising approach for advanced adsorption and separation processes. Mercury contamination, particularly at high concentrations, poses a serious threat to water safety due to its extreme toxicity, persistence, and bioaccumulation.^[^ ^60^ ^]^ Conventional mercury adsorbents often struggle with efficiency under high mercury loads, leading to secondary pollution and limited reusability. Our sulfur‐functionalized ZIF‐8 demonstrates exceptional Hg^2+^ ion adsorption capacity and recyclability, leveraging strong Hg‐S interactions for efficient mercury removal.

## 2 Results and Discussion

**Figure 1:**
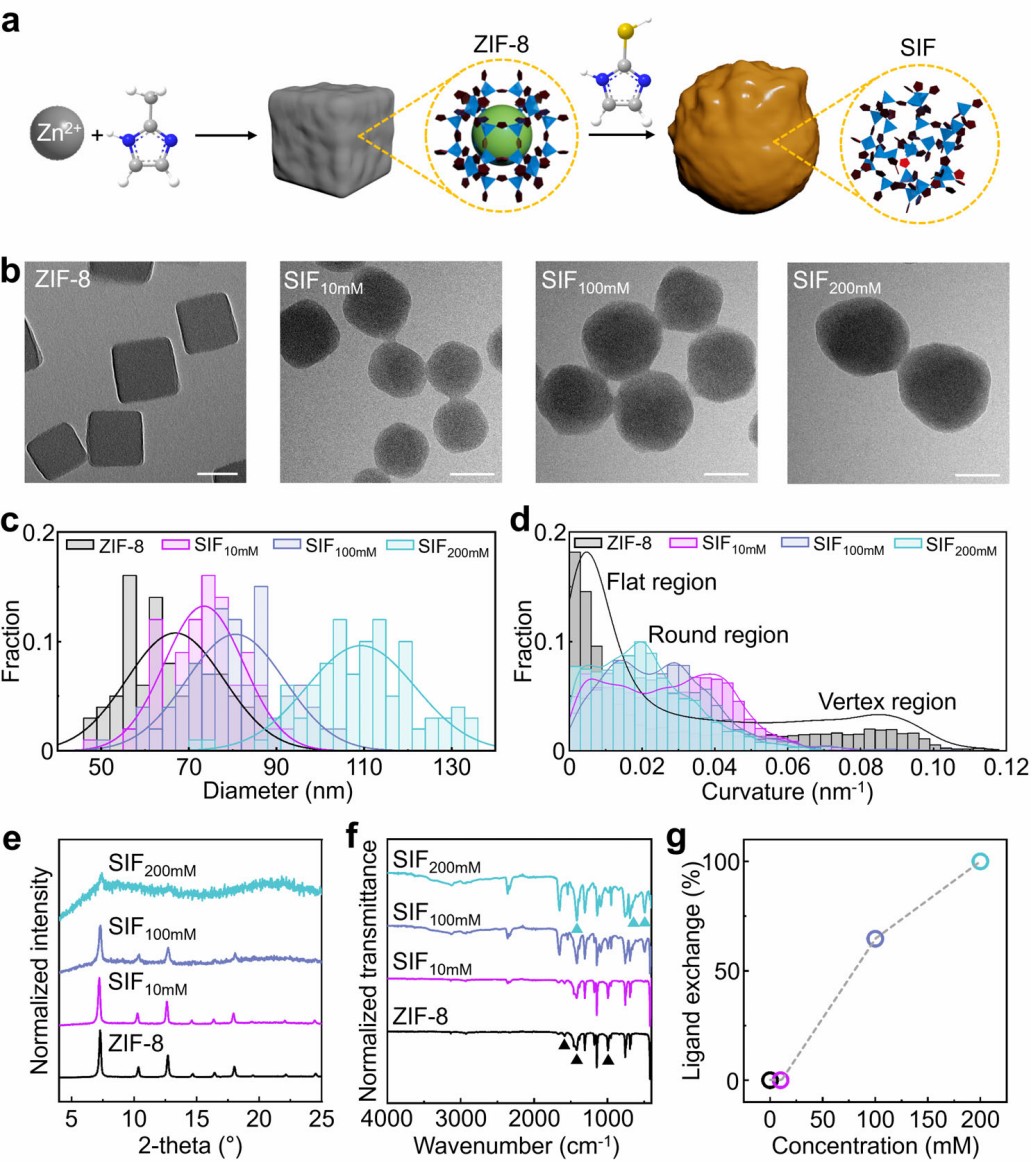
a) Schematic illustration of the formation of ZIF‐8 nanocrystals and the SALE process to SIF (gray spheres: carbon atoms, blue spheres: nitrogen atoms, white spheres: hydrogen atoms, and yellow spheres: sulfur atoms). The crystalline porous structure is shown for ZIF‐8, while the amorphous porous structure is depicted for SIF. b) Representative TEM images of ZIF‐8, SIF_10mm_, SIF_100mm_, and SIF_200mm_. c) Particle size distribution histograms for ZIF‐8 (dark gray bars), SIF_10mm_ (magenta bars), SIF_100mm_ (light navy bars), and SIF_200mm_ (light blue bars). d) Curvature distribution histograms for ZIF‐8 (dark gray bars), SIF_10mm_ (magenta bars), SIF_100mm_ (light navy bars), and SIF_200mm_ (light blue bars). Three regions are categorized based on the curvature value: flat region (left), round region (middle), and vertex region (right). e) XRD patterns of ZIF‐8 (black line), SIF_10mm_ (magenta line), SIF_100mm_ (light navy line), and SIF_200mm_ (light blue line). f) IR spectra of ZIF‐8 (black line), SIF_10mm_ (magenta line), SIF_100mm_ (light navy line), and SIF_200mM_ (light blue line). The characteristic peak positions are marked with triangular symbols (black for 2‐methylimidazole and light blue for 2‐mercaptoimidazole) g) Plot of ligand exchange ratio as a function of 2‐mercaptoimidazole concentration (black circle: 0 mm, magenta circle: 10 mm, light navy circle: 100 mm, and light blue circle: 200 mm). The ligand exchange ratio was estimated using the peak areas from the ^1^H NMR spectra. The dotted line serves as a visual guide. Scale bar: 50 nm.

ZIF‐8 nanocrystals with a cubic shape and high uniformity were synthesized by reacting zinc nitrate with 2‐methylimidazole in the presence of hexadecyltrimethylammonium bromide (CTAB) using a modified method.^[^ ^61^, ^62^ ^]^ CTAB regulates the growth of ZIF‐8 crystals, restricting crystal facet development and yielding a cubic morphology with particle sizes below 100 nm.^[^ ^63^, ^64^, ^65^ ^]^ Among the various possible ZIF‐8 shapes, we selected the cubic form because it enables more reliable detection of morphological changes, such as curvature variations, during image analysis. The nanocrystals had an average edge length of 66.9 ± 11.1 nm and were composed of {100} crystal facets. SALE of the ZIF‐8 nanocrystals was conducted by dispersing them in a methanolic solution with 2‐mercaptoimidazole at varying concentrations ranging from 10 to 200 mm (**Figure** **1**a), resulting in a framework containing thiolated organic linkers, referred to as SIF. Transmission electron microscopy (TEM) revealed that the cubic morphology of the ZIF‐8 nanocrystals gradually became rounded with increasing concentrations of 2‐mercaptoimidazole, eventually forming a fully spherical shape at 200 mm after the SALE process (Figure 1b and Figure S1, Supporting Information). In addition, an increase in the size of the ZIF‐8 nanocrystals was observed with higher concentrations of 2‐mercaptoimidazole, 73.4 ± 9.0 nm for SIF_10mm_, 80.7 ± 11.2 nm for SIF_100mm_, and 109.3 ± 12.4 nm for SIF_200mm_ (Figure 1c). Compared to the original edge length of the ZIF‐8 nanocrystals, the size was increased by 10% for SIF_10mm_, 21% for SIF_100mm_, and 63% for SIF_200mm_. A one‐way ANOVA confirmed that the differences in average particle size among the four treatment groups are statistically reliable (Figure S2, Supporting Information).

The degree of roundness of the ZIF‐8 nanocrystals was quantified using the open‐source software ImageJ and our customized MATLAB code,^[^ ^61^, ^66^ ^]^ which calculated the local curvature value for each particle contour based on TEM images (Figure 1d). The particle contours were color‐coded according to the extent of the local curvature value, defined as the inverse of the radius of the best‐fitted circle (Figures S3–S6, Supporting Information). High curvature values larger than 0.06 nm^−1^ were primarily observed around particle vertices in the ZIF‐8 nanocrystals before the SALE process, while the curvature distribution of the particle contours showed a dominant fraction near 0 nm^−1^, reflecting that most of the ZIF‐8 nanocrystals consisted of flat edges. As the concentration of 2‐mercaptoimidazole increased, the fraction near 0 nm^−1^ decreased to 72.9% for SIF_10mm_, 81.0% for SIF_100mm_, and 57.5% for SIF_200mm_, with a corresponding increase in higher curvature values, indicating a transition from flat edges to a rounded surface contour. We supposed that the SALE with 2‐mercaptoimidazole induced an abrupt surface reaction and structural reconstruction of the ZIF‐8 nanocrystals due to the high chemical reactivity of the exchange linker.

The crystallinity of the ZIF‐8 nanocrystals before and after SALE was analyzed using X‐ray diffraction (XRD), as shown in Figure 1e. ZIF‐8 exhibited characteristic peaks at 7.3°, 10.3°, and 12.7°,^[^ ^67^ ^]^ confirming the robustness of their porous structure. SIF_10mm_ also displayed characteristic peaks at the same angles; however, the full width at half maximum (FWHM) of the peaks at 7.3° broadened from 0.17 to 0.19, and the background noise signal was relatively higher. For SIF_100mm_, this trend became more pronounced, with significantly broadened peaks (FWHM = 0.23 at 7.3°) and a clearly elevated background noise level. In the case of SIF_200mm_, only a weak peak at 7.3° was observed, while all other peaks disappeared, indicating a complete loss of the crystalline structure of ZIF‐8. These findings suggest that the original porous framework of ZIF‐8 gradually collapses during the SALE process.

The molecular structures of the ZIF‐8 nanocrystals before and after the SALE were analyzed using infrared (IR) spectroscopy (Figure 1f). In the IR spectrum of ZIF‐8, four distinct peaks were observed at 420 cm^−1^ (Zn‐N stretching mode), 995 cm^−1^ (methyl C─H bending mode), 1421–1458 cm^−1^ (imidazole ring stretching mode), and 1585 cm^−1^ (C═N stretching mode), corresponding to the coordination of 2‐methylimidazole with zinc ions in the ZIF‐8 framework.^[^ ^68^, ^69^, ^70^ ^]^ As the concentration of 2‐mercaptoimidazole increased, notable changes were observed at 492, 949, and 1421–1458 cm^−1^. These characteristic peaks were identified as the S‐S stretching mode at 492 cm^−1^, the C‐S stretching mode at 736 cm^−1^, and the imidazole ring stretching mode at 1458 cm^−1^, with varying transmittance ratios due to the presence of 2‐methylimidazole.^[^ ^71^ ^]^ The broad peak ≈3141 cm^−1^ was attributed to hydrogen bonding between organic linkers (Figure S7, Supporting Information).^[^ ^72^ ^]^ The incorporation of 2‐mercptomidazole was also confirmed by the increases in such vibration modes. The signal at 492 cm^−1^ became prominent in SIF_100mM_ and SIF_200mM_, indicating that the structural transition process involved not only linker exchange but also thiol oxidation to disulfides.^[^ ^73^ ^]^

To quantify the fraction of exchanged 2‐mercaptoimidazole within the framework, NMR spectroscopy was conducted (Figure 1 g and Figures S8–S11, Supporting Information). NMR analysis determined the relative proportions of the original 2‐methylimidazole and exchanged 2‐mercaptoimidazole ligands within the framework (Table S1, Supporting Information). The proton peak corresponding to the methyl group of 2‐methylimidazole in ZIF‐8 appeared at 2.1 ppm, and that for the protons in the imidazole ring was measured at 6.8 ppm (Figure S8, Supporting Information). The ratio of the peak area (3 protons) at 2.1 ppm to that (2 protons) at 6.8 ppm was calculated as 1.5 for ZIF‐8, which was the same in SIF_10mM_ and SIF_100mM_, except for SIF_200mM_ which did not contain 2‐methylimidazole. The proton peak in the imidazole ring of 2‐mercaptoimidazole appears at 6.8–7.1 ppm with a slightly higher chemical shift than that in 2‐methylimidazole. The ratio of the peak area at 6.8 ppm to that at 7.1 ppm gradually increased from 0 for ZIF‐8 to 1.8 for SIF_100mM_ as the exchange ratio of 2‐mercaptoimidazole increased. Furthermore, SIF_200mM_ showed no peaks at 2.1 ppm and 6.8 ppm, indicating the complete absence of 2‐methylimidazole (Figures S9–S11, Supporting Information). Based on this analysis, the exchange ratio of 2‐mercaptoimidazole was determined to be 0% for SIF_10mM_, 64.6% for SIF_100mM_, and 100% for SIF_200mM_ (Figure 1g).

**Figure 2:**
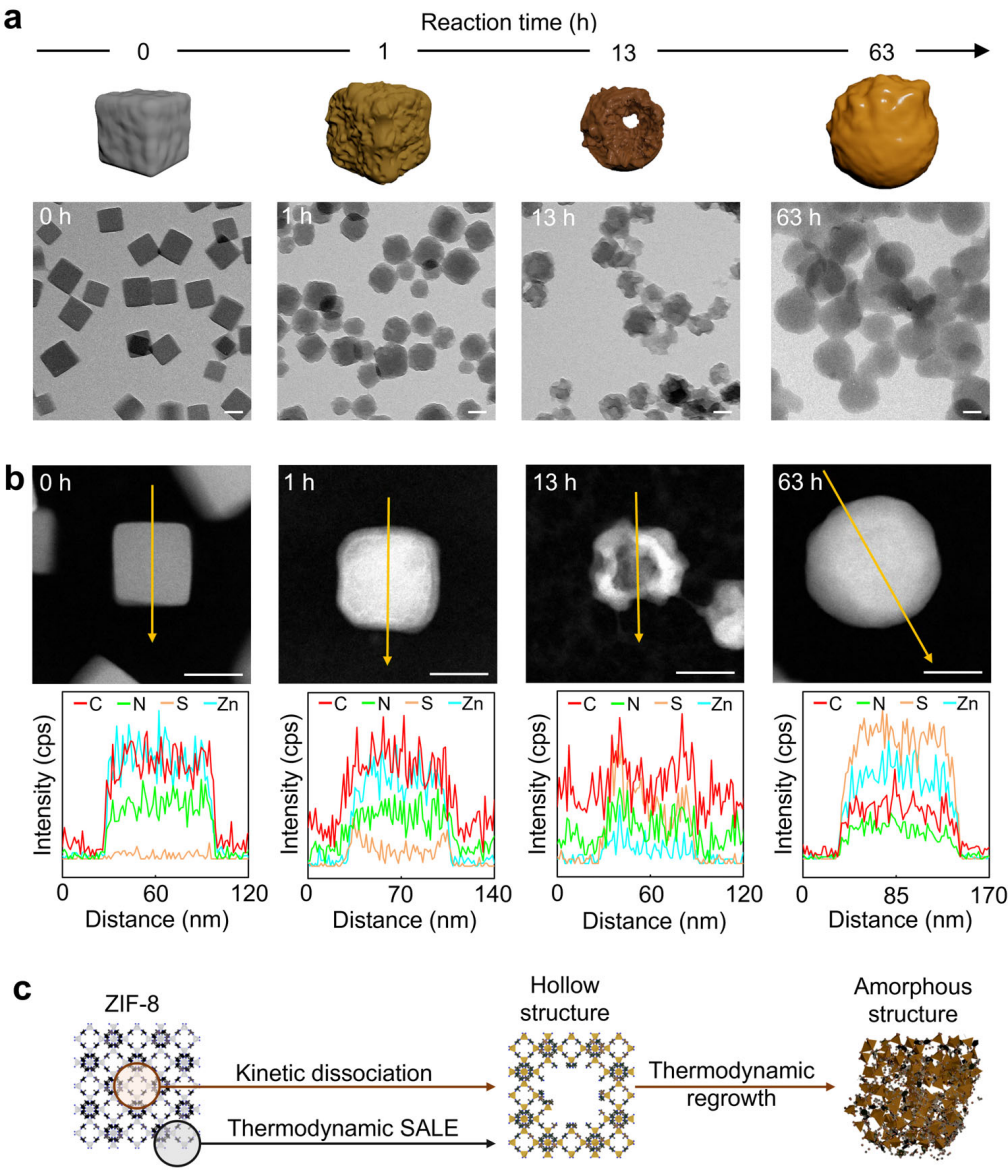
a) Representative TEM images and corresponding 3D structural representations illustrating the morphological reformation of SIF_200mM_ particles during the SALE process over time (from left to right: 0, 1, 13, and 63 h). b) STEM images with EDS line scans across SIF_200mM_ particles at different reaction times (from left to right: 0, 1, 13, and 63 h) to track elemental distributions (red: C, green: N, light brown: S, and light blue: Zn). c) Schematic illustration of the morphological transformation mechanism over time, suggested by kinetic dissociation and thermodynamic SALE processes. Scale bar: 50 nm.

The structural and compositional evolution of ZIF‐8 nanocrystals was investigated over 63 h at 200 mm 2‐mercaptoimidazole using TEM (**Figure** **2**a). Surface roughening was observed as early as 1 h into the 63‐h reaction. After 13 h, hollow structures formed as internal voids developed. The hollow structures then regrew into a spherical morphology, increasing in size. TEM elemental line scans revealed the nanocrystal composition (Figure 2b). Pristine ZIF‐8 nanocrystals showed a homogeneous Zn and N distribution, confirming uniform metal‐ligand coordination. After 1 h, the N signal decreased at the periphery, while the S signal slightly increased. At 13 h, the S signal dominated over N, while Zn was significantly depleted. At 63 h, S and N were uniformly distributed throughout the sphere, with Zn evenly dispersed. These findings suggest that ligand exchange with 2‐mercaptoimidazole induces ZIF‐8 etching, followed by reassembly of dissociated Zn^2+^ ions and 2‐mercaptoimidazole, ultimately forming spherical structures. Furthermore, the dissolution and reconstruction processes were optically monitored via transparency changes in the suspension (Figure S12, Supporting Information). The suspensions with 200 mm 2‐mercaptoimidazole remained transparent from the beginning of SALE to 13 h due to extensive dissolution and the formation of hollow structures. Thereafter, the suspension turned opaque pale yellow, indicating the reconstruction of amorphous SIF.

We propose that the morphological transition is driven by two distinct pathways: (1) a thermodynamic SALE process enabling the ideal ligand exchange between 2‐methylimidazole and 2‐mercaptoimidazole in ZIF‐8 and (2) a kinetic dissociation process leading to structural deformation (Figure 2c). 2‐Mercaptoimidazole can undergo an exchange reaction with the imidazole linkers from the surface of the framework. This exchange reaction can be categorized as a thermodynamic process competing with the kinetic dissociation induced by the proton‐driven ZIF‐8 collapse. As 2‐mercaptoimidazole acts as a weak acid in solution and provides protons^[^ ^48^ ^]^ they facilitate the dissociation of Zn‐imidazole bonds in the framework, triggering the structural collapse. In particular, owing to their high mobility, protons readily diffuse into the pores of ZIF‐8, accelerating internal collapse more rapidly than surface degradation, where ligand exchange occurs simultaneously. This results in the formation of an intermediate hollow structure. Furthermore, 2‐mercaptoimidazole coordinates with the Zn^2+^ ions released via the kinetic dissociation process, undergoing thermodynamic regrowth through recombination inside and outside the hollow framework. Ultimately, this process yields an amorphous spherical structure, where Zn^2+^ ions and 2‐mercaptoimidazole coexist in a coordinated state.

To predominantly drive the thermodynamically favored ligand exchange, we introduced aprotic polar solvents to suppress the kinetic dissociation process. Aprotic polar solvents were expected to reduce the proton dissociation of 2‐mercaptoimidazole, thereby inhibiting the kinetic dissociation pathway. In our ligand exchange experiments, we initially used methanol (MeOH) as the solvent, which is classified as a strongly protic polar solvent. To enhance aprotic characteristics, we employed tetrahydrofuran (THF), acetone (ACE), isopropanol (IPA), and ethanol (EtOH) as alternative solvents and examined their effects on ligand exchange and the morphological transformation of ZIF‐8 nanocrystals. It is noted that the ligand concentration of 50 mm was used due to the solubility limitations of 2‐mercaptoimidazole in aprotic polar solvents such as THF.

**Figure 3:**
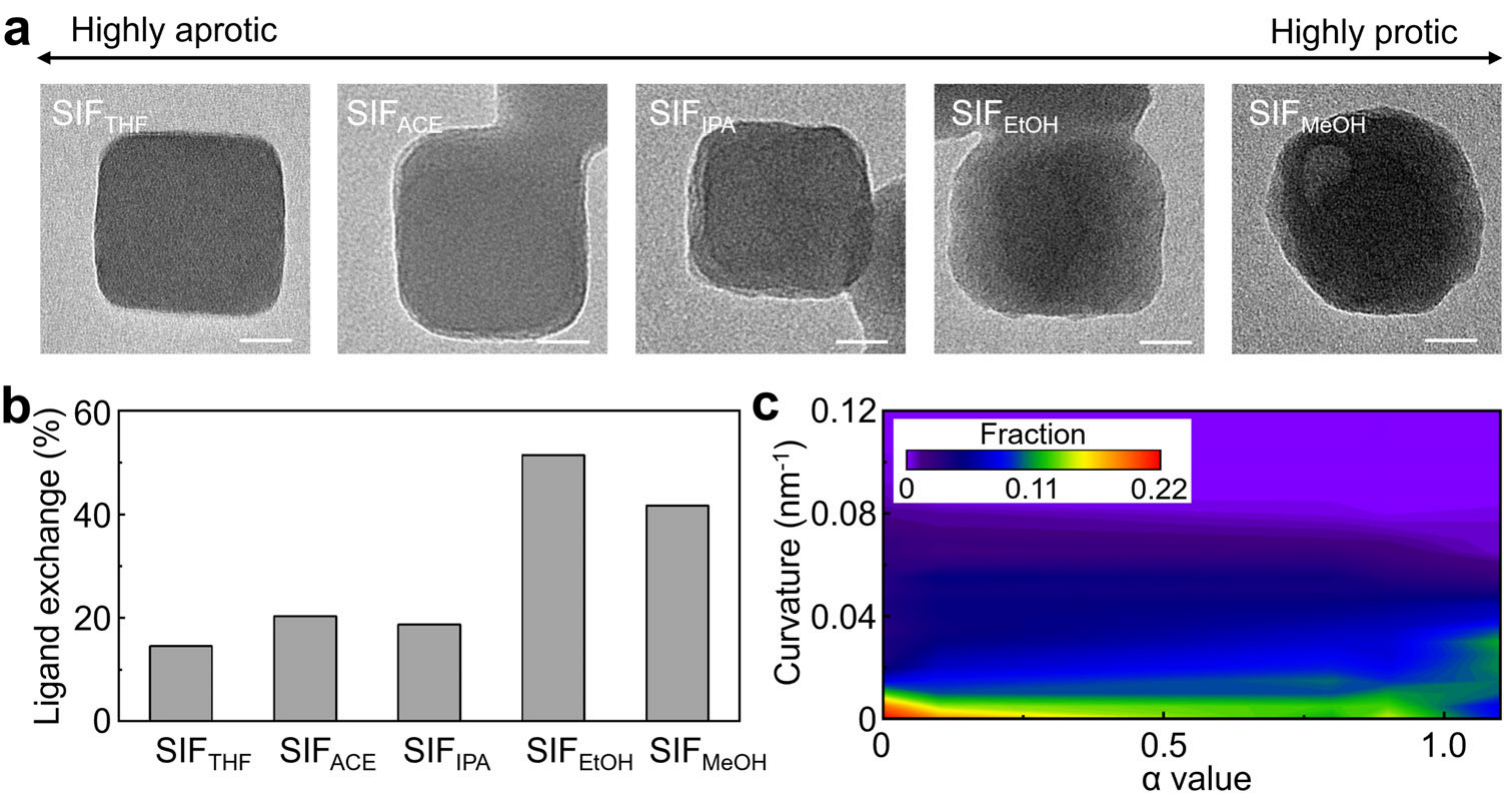
a) Representative TEM images of SIF_THF_, SIF_ACE_, SIF_IPA_, SIF_EtOH_, and SIF_MeOH_ obtained after 63 h of SALE in 50 mm 2‐mercaptoimidazole using different solvents (THF, ACE, IPA, EtOH, and MeOH). b) Ligand exchange ratios of SIF_THF_, SIF_ACE_, SIF_IPA_, SIF_EtOH_, and SIF_MeOH_, estimated from the peak areas in the ^1^H NMR spectra. c) Correlation map showing the curvature distribution as a function of the HBD values of the solvents. Scale bar: 20 nm.

TEM images (**Figures** **3**a and S13, Supporting Information) confirmed that when highly aprotic solvents, such as THF and ACE, were used, the morphology of ZIF‐8 nanocrystals remained nearly intact after ligand exchange. However, as the aprotic nature decreased—from THF to MeOH—noticeable morphological changes occurred in ZIF‐8, and its surface contour became more rounded. Furthermore, XRD analysis confirmed that the intensity and pattern of the characteristic peaks corresponding to ZIF‐8 were less affected in more aprotic solvents, whereas protic solvents introduced a lower peak intensity (Figure S14, Supporting Information). This suggests that the crystalline structure of ZIF‐8 is better preserved in aprotic environments, likely due to reduced framework etching caused by protons from 2‐mercaptoimidazole. To further investigate this effect, the ligand exchange ratio with 2‐mercaptoimidazole in different solvents was estimated using NMR analysis (Figures S15–S19, Supporting Information). The exchange ratios were determined to be 14.5% in SIF_THF_, 20.3% in SIF_ACE_, 18.7% in SIF_IPA_, 51.5% in SIF_EtOH_, and 41.7% in SIF_MeOH_ (Figure 3b), demonstrating that the ligand exchange ratio decreases as the aprotic nature of the solvent increases.

We analyzed the correlation between ligand exchange extent and morphological transformation with respect to hydrogen bond donor (HBD) value, integrating NMR spectra and curvature analysis data obtained for SIF in various solvents (Figure 3c and Figures S20–S24, Supporting Information). The HBD value, represented by the α parameter in the Kamlet‐Taft scale, measures the ability of a solvent to donate hydrogen bonds.^[^ ^74^ ^]^ Protic solvents exhibit high α values due to labile protons, whereas aprotic solvents have low or negligible α values, indicating weak hydrogen bond donation (α^THF^: 0.0, α^ACE^: 0.1, α^IPA^: 0.8, α^EtOH^: 0.9, and α^MeOH^: 1.1).^[^ ^75^ ^]^ The correlation color map revealed that as the α value decreases, the distribution of curvatures near 0 nm^−1^ increases (Figure 3c). Conversely, at higher α values, the distribution near 0 nm^−1^ decreases, while curvatures in the range of 0 to 0.04 nm^−1^ become more dominant. This suggests that higher α values are associated with more pronounced morphological changes in SIF particles. For the ligand exchange ratio, it showed that the exchange ratio stabilizes ≈20% as the α value increases up to 0.8. Beyond this threshold, the ligand exchange ratio rises above ≈40%. These findings indicate that aprotic solvents suppress proton release from 2‐mercaptoimidazole during ligand exchange, thereby minimizing morphological changes through the kinetic dissolution pathway. However, the inhibition of the thermodynamic SALE process also leads to a lower ligand exchange ratio. This is likely due to the reduced mobility of 2‐mercaptoimidazole in aprotic solvents, along with its decreased reactivity with the polar ZIF‐8 surface, which contains a relatively high density of uncoordinated sites.

**Figure 4:**
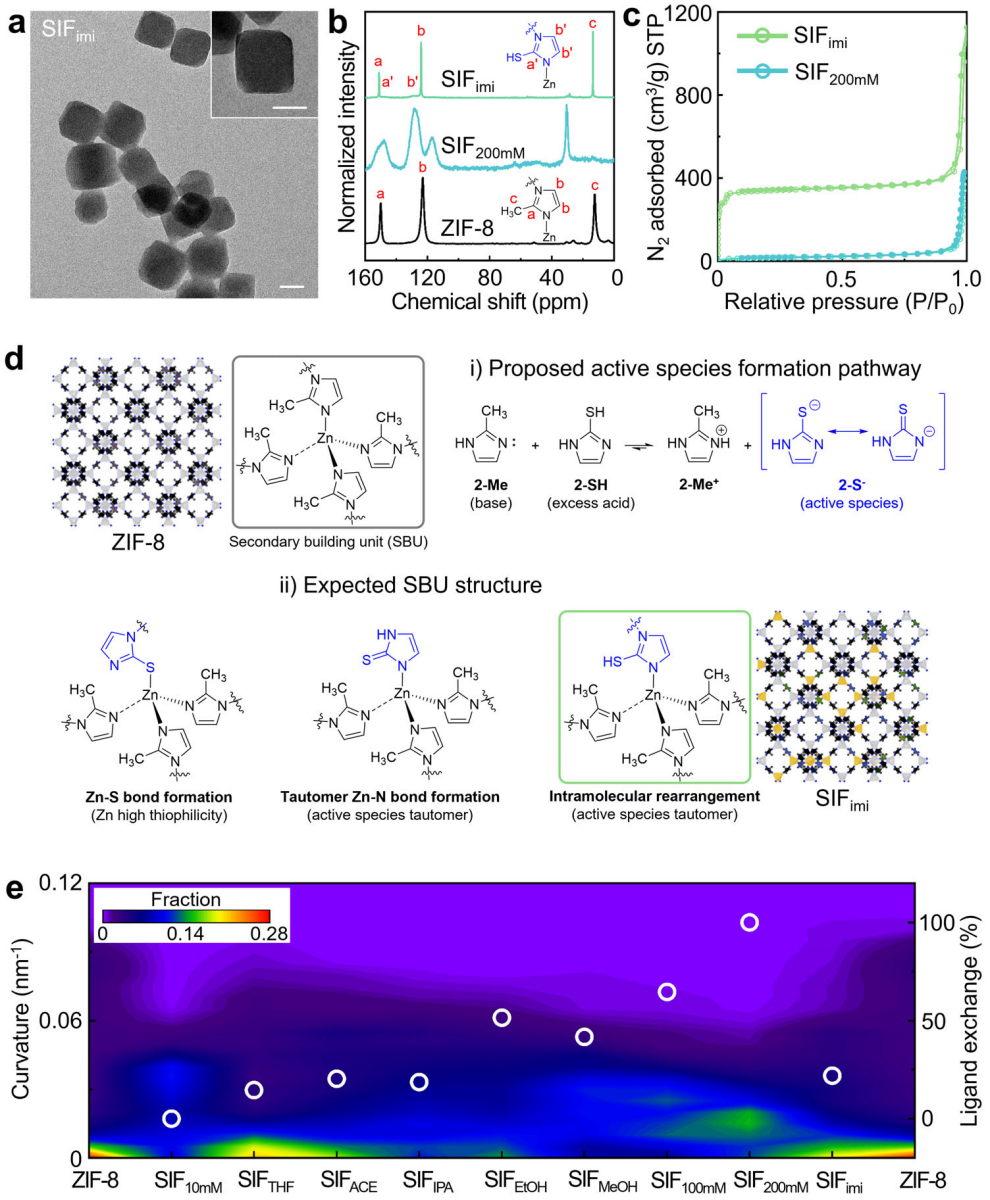
a) Representative TEM images of SIF_imi_. b) Solid‐state ^13^C NMR spectra of ZIF‐8 (black line), SIF_200mM_ (light blue line), and SIF_imi_ (light green line). c) N_2_ physisorption isotherms at 77 K for SIF_200mM_ (light blue line) and SIF_imi_ (light green line). d) Schematic illustration of the ligand additive‐stabilized SALE mechanism for SIF_imi_. The kinetic dissociation process is suppressed in favor of the thermodynamic SALE process. e) Correlation map showing the curvature distribution and ligand exchange ratio for various materials (from left to right: SIF_10mM_, SIF_THF_, SIF_ACE_, SIF_IPA_, SIF_EtOH_, SIF_MeOH_, SIF_100mM_, SIF_200mM_, and SIF_imi_). The white circles indicate the ligand exchange values for each material. The curvature distribution of ZIF‐8 is provided for comparison. Scale bar: 50 nm.

To suppress kinetic dissolution while promoting the thermodynamic SALE process, we introduced 2‐methylimidazole into the ligand exchange reaction, resulting in SIF particles with moderate morphological changes, referred to as SIF_imi_. The stabilizing effect was supported by TEM analysis, which showed that the SIF structure maintained its original form (**Figure** **4**a). The particle surface curvature analysis showed a significant distribution near 0 nm^−1^, indicating minimal deformation (Figure S25, Supporting Information). In contrast, without the addition of 2‐methylimidazole, the cubic ZIF‐8 nanocrystals adopted a spherical morphology in SIF_200mM_.

The ^13^C solid‐state NMR spectra provide clear evidence of structural differences among ZIF‐8, SIF_200mM_, and SIF_imi_, highlighting the influence of ligand substitution and amorphization on the local chemical environment (Figure 4b). For ZIF‐8, well‐defined peaks corresponding to C2 of imidazole (151 ppm), C4/C5 of imidazole (123 ppm), and the methyl group (13–14 ppm) were observed, indicating a highly ordered crystalline framework based on 2‐methylimidazolate coordination with Zn^2+^. In contrast, the spectrum of SIF_200mM_ exhibits significant peak broadening and chemical shift variations, particularly in the C2 and C4/C5 regions, consistent with the transition to an amorphous phase composed of Zn^2+^ and 2‐mercaptoimidazole. In the case of SIF_imi_, the incorporation of 2‐mercaptoimidazole into the ZIF‐8 framework results in the emergence of new peaks (C2′: 128 ppm and C4″/C5″: 148 ppm) next to C2 and C4/C5 of imidazole. Compared to SIF_200mM_, the sharper signals of SIF_imi_ indicate that it retains a more ordered coordination environment, preserving the original ZIF‐8 structure.

The porosity and crystallinity of SIF_imi_ were analyzed using Brunauer‐Emmett‐Teller (BET) surface area measurement and XRD. SIF_imi_ retained a surface area of 1014 m^2^ g^−1^, comparable to ZIF‐8 (Figure S26, Supporting Information), whereas SIF_200mM_, formed without 2‐methylimidazole, exhibited a sharp decline to 52 m^2^ g^−1^, indicating a significant reduction in microporosity (Figure 4c). In addition, we confirmed that the surface area of SIF_10mM_ (987 m^2^ g^−1^) approached that of pristine ZIF‐8, whereas it significantly decreased in SIF_100mM_ (100 m^2^ g^−1^) in Figure S27 and Table S2 (Supporting Information). The pore size distribution further revealed that the fraction of micropores smaller than 1.5 nm disappeared in SIF_100mM_ and SIF_200mM_ (Figure S28, Supporting Information). These results suggest that unstable ligand exchange leads to a loss of porosity, while successful ligand exchange, as in SIF_imi_, helps preserve the porous structure of ZIF‐8. This indicates that the presence of 2‐methylimidazole plays a key role in preventing structural collapse and maintain intrinsic porosity. XRD confirmed that despite exposure to a high ligand concentration (200 mm), SIF_imi_ maintained peak patterns and intensities similar to ZIF‐8 (Figure S29, Supporting Information), indicating minimal kinetic dissolution during ligand exchange. IR spectroscopy further supported this (Figure S30, Supporting Information), as the hydrogen bonding of randomly arranged ligand peaks, observed in the range of 2460–3618 cm^−1^ and prominent in SIF_200mM_, were significantly reduced in SIF_imi_. In addition, SIF_imi_ exhibited high structural stability in solvents of varying polarity (Figure S31, Supporting Information). These findings highlight the effectiveness of our ligand additive‐stabilized SALE strategy in maintaining the ZIF‐8 framework while enabling extensive ligand exchange.

Since free thiol groups play a critical role in Hg^2+^ coordination, minimizing disulfide formation is essential for enhancing Hg adsorption performance. To better understand the chemical transformations associated with ligand exchange and their impact on adsorption properties, we investigated the formation of disulfide species. Disulfide formation was first confirmed by the appearance of characteristic S‐S stretching bands in the IR spectra, prompting us to perform X‐ray photoelectron spectroscopy (XPS) S 2p analysis to quantify the relative contents of thiol and disulfide (Figure S32, Supporting Information). Deconvolution of the S 2p envelope (with a fixed 2:1 area ratio for S 2p_3/2_:S 2p_1/2_) revealed that SIF_10mM_ contains ≈12% disulfide, whereas SIF_100mM_ and SIF_200mM_ show ≈50% and ≈56% disulfide, respectively. In contrast, SIF_imi_ exhibits a lower disulfide fraction of ≈30%, demonstrating that the ligand additive suppresses R‐SH coupling to R‐S‐S‐R. These XPS results confirm and quantify the IR‐observed conversion of thiol to disulfide and highlight the effectiveness of excess ligand in inhibiting disulfide formation during SALE, thereby helping preserve active thiol sites for Hg capture.

We supposed that 2‐methylimidazole in solution acts as a proton acceptor for the dissociated proton from 2‐mercaptoimidazole, thus stabilizing the framework and directing the exchange process toward a thermodynamically favorable state (Figure 4d). The thiol group of 2‐mercaptoimidazole (pK_a_ 10.3) exhibits a lower pK_a_ than the pyrrole nitrogen of 2‐methylimidazole (pK_a_ 18.6), making it the preferred proton donor.^[^ ^76^ ^]^ The released proton can be accepted by the pyridine nitrogen of 2‐methylimidazole, facilitating proton exchange and stabilizing the coordination environment. As a result, 2‐mercaptoimidazole can exist in equilibrium between two tautomeric forms: 2‐thiolateimidazole and 2‐thioneimidazolate. Although 2‐thiolateimidazole can coordinate with Zn through Zn‐S bonding, forming an active thiophilic Zn species, the structural integrity and BET surface area of SIF_imi_ remain consistent with the pristine ZIF‐8. This suggests that Zn‐N bonding is dominant, likely due to the stabilization effect of 2‐thioneimidazolate. Finally, 2‐thioneimidazolate may undergo tautomerization to 2‐thiolate upon proton acceptance from an adjacent N‐H group, maintaining the Zn‐N coordination.

We systematically examined how exchange ligand concentration, solvent type, and ligand additive influence the morphological transformation and ligand exchange ratio of ZIF‐8 nanocrystals during the SALE process (Figure 4e). At higher ligand concentrations, ZIF‐8 nanocrystals exhibited increased morphological deformation, as indicated by deviations in curvature values from 0 nm^−1^, along with weakened crystallinity and porosity. Although the ligand exchange ratio increased from 0% (SIF_10mM_) to 100% (SIF_200mM_), the resulting coordination environment suggested a disruption of the intrinsic microporous structure. Notably, introducing an aprotic solvent effectively stabilized the ZIF‐8 framework, as evidenced by a greater proportion of curvature values remaining near 0 nm^−1^ in SIF_THF_ and SIF_ACE_, despite the same ligand concentration in SIF_MeOH_. This structural stabilization was accompanied by a reduction in the ligand exchange ratio to ≈20%. To further enhance stability while maintaining a high ligand exchange ratio, we introduced 2‐methylimidazole in the condition of 200 mm 2‐mercaptoimidazole. Under this condition, the ZIF‐8 structure remained well‐preserved, with a greater proportion of curvature values converging near 0 nm^−1^. The ligand exchange ratio also reached ≈22% (Figure S33, Supporting Information). The resulting SIF_imi_ exhibited high porosity and a large surface area, with incorporated sulfur sites in the microporous framework, making it a promising candidate for metal ion adsorption.

**Figure 5:**
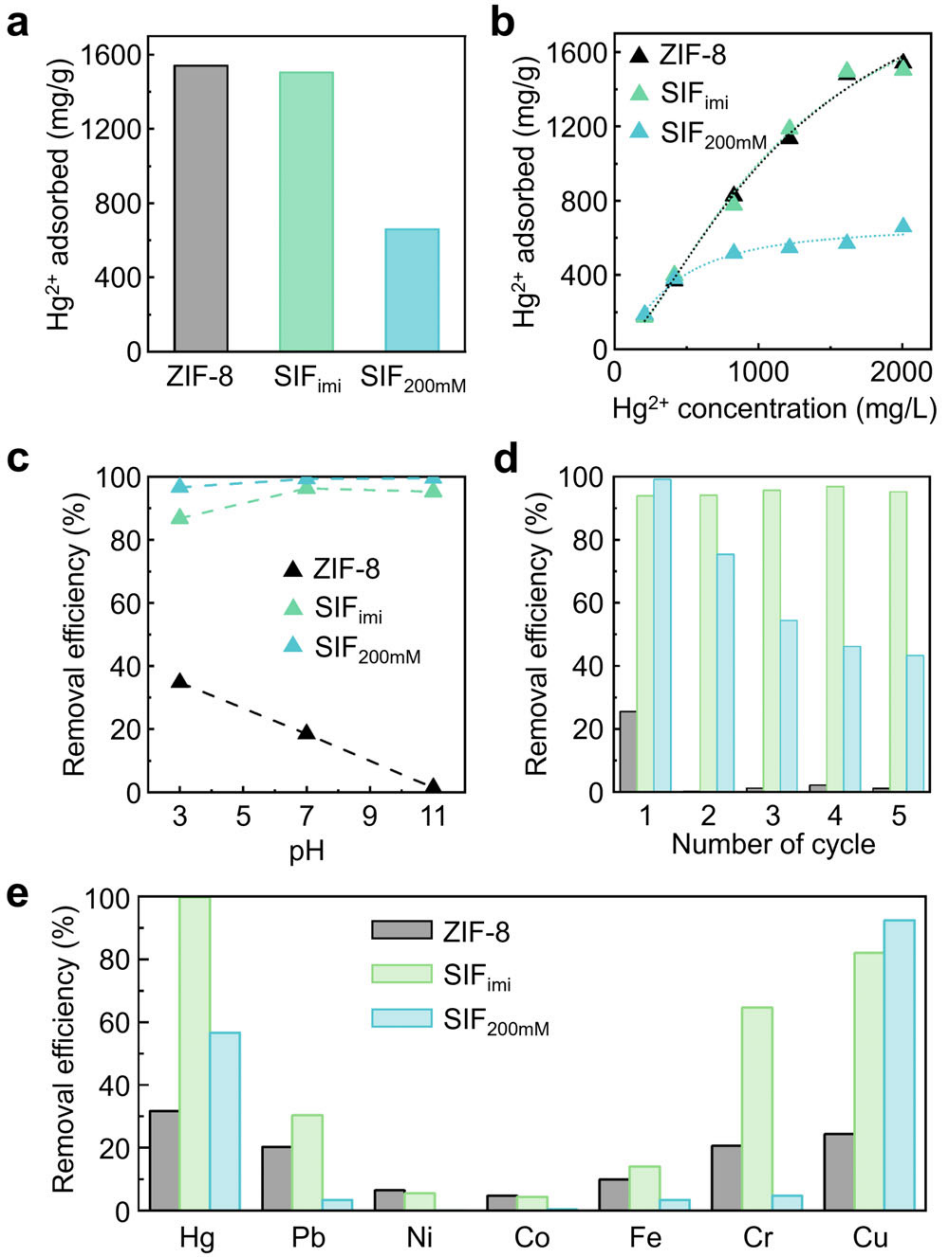
a) Adsorption capacity of Hg^2+^ ions (10 mm) by ZIF‐8 (gray bar), SIF_imi_ (light green bar), and SIF_200mM_ (light blue bar) during a single adsorption cycle. b) Hg^2+^ adsorption capacity as a function of initial Hg^2+^ concentration by ZIF‐8 (black triangle), SIF_imi_ (light green triangle), and SIF_200mM_ (light blue triangle), fitted to the Langmuir adsorption model. c) Hg^2+^ removal efficiency over different pH conditions at 10 ppm HgCl_2_. d) Hg^2^⁺ removal efficiency over five consecutive adsorption‐desorption cycles at an initial Hg^2+^ concentration of 10 ppm. e) Multi‐ion adsorption selectivity test for seven metal ions (Hg^2+^, Pb^2+^, Ni^2+^, Co^2+^, Fe^3+^, Cr^3+^, and Cu^2+^: 10 ppm each) in a single batch experiment.

We compared the mercury ion adsorption performance of three materials: (i) SIF_imi_, which is highly porous and functionalized with thiol groups, (ii) SIF_200mM_, which has a high ligand exchange ratio but poor porosity, and (iii) ZIF‐8 as a control (**Figure** **5**). Mercury ion adsorption experiments were conducted using a 10 mm aqueous solution of mercury chloride (2005 ppm HgCl_2_). Both ZIF‐8 and SIF_imi_ exhibited significant Hg^2+^ ion adsorption (removal capacity: 1540 mg g^−1^ for ZIF‐8 and 1504 mg g^−1^ for SIF_imi_), whereas SIF_200mM_ showed a significantly lower removal capacity of 659 mg g^−1^ (Figure 5a). In addition, adsorption isotherm analysis revealed that all three materials followed the Langmuir model rather than the Freundlich adsorption model (Figure 5b), indicating monolayer adsorption of Hg^2+^ ions on heterogeneous surfaces.^[^ ^77^ ^]^ Although ZIF‐8 lacks functional groups for strong chemical interactions with Hg^2+^ ions, Hg^2+^ ion adsorption in ZIF‐8 appears to be primarily driven by metal cation exchange due to the similar chemical properties of Zn^2+^ and Hg^2+^.^[^ ^78^ ^]^

Although the initial Hg^2+^ ion adsorption capacities of ZIF‐8 and SIF_imi_ were comparable, SIF_imi_ demonstrated significantly higher reusability over multiple cycles at 1444 ppm HgCl_2_ (Figure S34, Supporting Information). After five adsorption–desorption cycles, ZIF‐8 exhibited a substantial decrease in Hg^2+^ ion adsorption. This suggests that once Zn^2+^ sites were exchanged with Hg^2+^, further adsorption became limited. In contrast, SIF_imi_ maintained a high removal efficiency, retaining 52% of its Hg^2+^ ion adsorption capacity after five cycles. This stability can be attributed to the presence of the additional thiol functional groups, which serve as recyclable Hg^2+^ adsorption sites. On the other hand, SIF_200mM_ exhibited lower initial adsorption due to its amorphous structure and low porosity. After the third cycle, its Hg^2+^ adsorption capacity surpassed that of ZIF‐8. This could be attributed to the presence of thiol functional groups on the surface, which might have functioned as recyclable adsorption sites. The combination of high initial adsorption capacity and excellent reusability of Hg^2+^ ions makes SIF_imi_ an attractive option for mercury ion removal technologies (Table S3, Supporting Information).

To better simulate realistic environmental conditions, we evaluated the mercury adsorption performance of SIF_imi_, SIF_200mM_, and ZIF‐8 at a low concentration (10 ppm HgCl_2_) under different pH conditions (Figure 5c). SIF_imi_ exhibited outstanding performance, maintaining over 95% removal efficiency across the entire pH range and retaining 95% of its initial uptake capacity after five adsorption–desorption cycles (Figure 5d). The XRD patterns further confirmed the structural stability of SIF_imi_ (Figure S35, Supporting Information). SIF_200mM_ also maintained high removal efficiency (>95%) across the pH conditions, while it showed reduced reusability, retaining only 43% of its initial capacity after five cycles. It is likely due to lower structural robustness in SIF_200mM_. In contrast, ZIF‐8 exhibited poor Hg^2+^ removal efficiency (<19%) and minimal reusability (1% retention). The combination of structural robustness, consistent adsorption across a wide pH range, and superior reusability highlights SIF_imi_ as a highly viable candidate for real‐world mercury remediation under environmentally relevant conditions.

In addition, to assess competitive adsorption performance, we conducted selectivity tests in a multi‐ion system containing Hg^2+^, Pb^2+^, Ni^2+^, Co^2+^, Fe^3+^, Cr^3+^, and Cu^2+^ (each at an initial concentration of 10 ppm) (Figure 5e). SIF_imi_ exhibited the highest and most selective removal of Hg^2+^ (≈100% efficiency), clearly outperforming both SIF_200mM_ and ZIF‐8. On the other hand, SIF_200mM_ showed reduced selectivity toward Hg^2+^ (57%) compared to its performance in single‐ion systems, indicating that excess thiol groups may lead to non‐specific binding in complex ionic matrices. In addition to mercury, SIF_imi_ also exhibited appreciable selectivity for Cu^2+^ (82%) and Cr^3+^ (65%), while the uptake of other metal ions remained minimal. These results firmly establish SIF_imi_ as the most selective sorbent for mercury removal in complex aqueous environments.

To provide direct evidence of Hg‐S coordination, we performed XPS on the Hg 4f region of ZIF‐8, SIF_imi_, and SIF_200mM_ after adsorption of 10 ppm Hg^2+^ (Figure S36, Supporting Information). ZIF‐8 showed no detectable Hg signal, whereas SIF_imi_ and SIF_200mM_ exhibited distinct Hg 4f_7/2_ and 4f_5/2_ peaks at 100.47 and 104.47 eV, respectively (Table S4, Supporting Information), consistent with literature values for Hg‐S bonding.^[^ ^79^, ^80^ ^]^ In contrast, when samples were exposed to a higher Hg^2+^ concentration (2005 ppm), ZIF‐8 showed clear Hg signals with a single Hg 4f doublet at higher binding energies (Figure S37 and Table S4, Supporting Information), characteristic of Hg‐N coordination. In comparison, SIF_imi_ and SIF_200mM_ exhibited two distinct Hg 4f doublets: one at higher binding energy corresponding to Hg‐N, and a second at lower binding energy, indicative of Hg‐S coordination. These results confirm that thiol‐functionalized frameworks facilitate Hg‐S coordination, in contrast to unmodified ZIF‐8, which binds Hg exclusively through imidazole nitrogen. Under conditions of excessive Hg^2^⁺ loading, partial metal ion exchange between Hg^2+^ and Zn^2+^ may also occur.

To explain the enhanced Hg^2+^ uptake in SIF_imi_, we refer to prior density functional theory (DFT) studies on Hg‐S interactions and propose three representative binding modes (Figure S38, Supporting Information): (i) window‐site coordination at dense sulfur clusters, (ii) metal‐imidazole nitrogen exchange, and (iii) terminal binding to a single thiol group. These models provide a theoretical basis for interpreting our experimental results. DFT calculations report strong Hg^2+^ affinity for sulfur‐containing sites. For example, UiO‐66‐SH shows an adsorption energy of 48.9 kcal mol^−1^ at dual‐thiol sites,^[^ ^81^ ^]^ and UiO‐66‐BAT‐SH exhibits preferential binding to sulfhydryl groups with −1.94 eV.^[^ ^82^ ^]^ At imidazole nitrogen, a binding energy of −53.58 kcal mol^−1^ has been reported.^[^ ^51^ ^]^ These results support the coexistence of Hg‐S and Hg‐N coordination in SIF_imi_ and reinforce the role of thiol groups in enhancing mercury binding.

## 3 Conclusion

We have developed an efficient ligand exchange strategy for ZIF‐8 nanocrystals, introducing sulfur‐containing ligands as Lewis base active sites while minimizing morphological and structural alterations. By controlling the concentration of the exchange ligand, 2‐mercaptoimidazole, we investigated trends in morphological and structural transformations. Analysis of intermediates at various reaction times enabled us to elucidate the ligand exchange mechanism, which involves the kinetic dissolution of the ZIF‐8 structure induced by protons from 2‐mercaptoimidazole, followed by a thermodynamically driven SALE process. The correlation between ligand concentration, HBD solvent conditions, morphological stability, and ligand exchange efficiency was quantitatively analyzed using surface curvature measurements from image analysis and NMR spectroscopy. As a result, we identified the structural stabilization effect of ligand incorporation, which preserved the ZIF‐8 morphology while improving the ligand exchange ratio. The resulting sulfur‐functionalized ZIF‐8 exhibited exceptional Hg^2+^ adsorption performance, achieving a capacity of 1504 mg g^−1^, high removal efficiency across a wide pH range, excellent recyclability, and strong selectivity even under competitive multi‐ion conditions. This enhanced adsorption capacity highlights SIF_imi_ as a promising candidate for mercury removal, offering a durable and effective solution for mercury‐contaminated environments. Furthermore, our ligand additive‐stabilized SALE approach holds great potential for designing and applying functionalized MOFs for diverse applications.

## Conflict of Interest

The authors declare no conflict of interest.

## Supporting information



Supporting Information

## Data Availability

The data that support the findings of this study are available from the corresponding author upon reasonable request.
