# Supplementary material for: Sulfur‐Functionalized MOF via Ligand Additive‐Stabilized SALE for Efficient Hg2+ Ion Removal
Source: Small. 2025 Jul 9;21(35):2503637. doi: 10.1002/smll.202503637 (PMC12410910; doi:10.1002/smll.202503637)
Supplement: Supplementary file 1 — Supporting Information [file SMLL-21-2503637-s001.pdf]

NANO · MICRO
small

Supporting Information

for *Small*, DOI 10.1002/smll.202503637

Sulfur-Functionalized MOF via Ligand Additive-Stabilized SALE for Efficient Hg^{2+} Ion Removal

*Cheongwon Bae, Ho-Jun Cho, Ju Hyun Kim, Xiaohui Song and Juyeong Kim**

Supporting Information

Sulfur-Functionalized MOF via Ligand Additive-Stabilized SALE for Efficient Hg^{2+} Ion Removal

Cheongwon Bae,[a,b] Ho-Jun Cho,[a,b] Ju Hyun Kim,[c] Xiaohui Song[d,e] and Juyeong Kim[a,b,]*

[a]Department of Chemistry, Gyeongsang National University, Jinju 52828, South Korea

[b]Research Institute of Advanced Chemistry, Gyeongsang National University, Jinju 52828, South Korea

[c]Department of Chemistry, Dongguk University, Seoul 04620, South Korea

[d]School of Materials Science and Engineering, Hefei University of Technology, Anhui Province 230009, China

[e]Engineering Research Center of High Performance Copper Alloy Materials and Processing, Ministry of Education, Hefei University of Technology, Hefei 230009, China

*Corresponding author. Email: chris@gnu.ac.kr (J. Kim)

Materials and Methods

Figures S1–S38

Table S1–S4

References

Materials and Methods

Section 1. Chemicals

Zinc nitrate hexahydrate (98%, $Zn(NO_3)_2 \cdot 6H_2O$, Sigma-Aldrich), 2-methylimidazole (99%, $C_4H_6N_2$, Sigma-Aldrich), 2-mercaptoimidazole (98%, $C_3H_4N_2S$, Sigma-Aldrich), cetyltrimethyl ammonium bromide (CTAB, 99%, $C_{19}H_{42}BrN$, Sigma-Aldrich), methyl alcohol (MeOH, 99.5%, CH_3OH, Daejung), tetrahydrofuran (THF, >99.0%, C_4H_8O, Daejung), acetone (ACE, >99.8%, C_3H_6O, Daejung), anhydrous ethyl alcohol (EtOH, 99.9%, C_2H_5OH, Samchun) iso-propyl alcohol (IPA, 99.9%, $(CH_3)_2CHOH$, Daejung), mercury chloride (99.5 %, $HgCl_2$, Thermo Scientific), lead chloride (98%, $PbCl_2$, Duksan), copper chloride (≥98.0%, $CuCl_2$, Sigma-Aldrich), nickel nitrate hexahydrate (94.5–105.5%, $Ni(NO_3)_2 \cdot 6H_2O$, Sigma-Aldrich), chromium nitrate nonahydrate (99%, $Cr(NO_3)_3 \cdot 9H_2O$, Sigma-Aldrich), cobalt chloride hexahydrate (99.9%, $CoCl_2 \cdot 6H_2O$, Thermo Scientific), iron chloride hexahydrate (98.0–102%, $FeCl_3 \cdot 6H_2O$, Sigma-Aldrich), and thiourea (98%, N_2H_4CS, Junsei) were purchased and used without further purification. Deionized water (18.2 MΩ·cm at 25 °C) purified by a Merck Millipore Direct Q3 UV Water Purification System was used for all washing and aqueous solution preparation. All glassware was cleaned with 1 M HCl, thoroughly rinsed with DI water and dried immediately before use.

Section 2. Synthesis of ZIF-8 and SALE to SIF

2.1. ZIF-8

ZIF-8 nanocrystals were prepared according to a literature method with modifications.[1] Aqueous solutions of 24 mM $Zn(NO_3)_2 \cdot 6H_2O$ and 1.32 M 2-methylimidazole were separately prepared in a 200 mL Erlenmeyer flask at room temperature. An aqueous solution of 50 mM CTAB was prepared in a 20 mL scintillation vial at 50°C under stirring. Then, 2 mL of the CTAB solution was added to 100 mL of the 2-methylimidazole solution, which was stirred at 500 rpm for 5 min at room temperature. The $Zn(NO_3)_2 \cdot 6H_2O$ solution (100 mL) was then injected into the mixture while stirring at 500 rpm. The solution was stirred for 5 min and then left undisturbed for 3 h. The resulting milky mixture was divided into ~35 mL portions and transferred into 50 mL conical tubes. The product solution was centrifuged at 9000 rpm for 30 min, and the supernatant was discarded. MeOH was added to the residue, and each residue was dispersed and washed with ~35 mL of MeOH. The washing and dispersion process was repeated. Finally, the ZIF-8 nanocrystals were stored in 200 mL of MeOH in an Erlenmeyer flask.

2.2. SALE of ZIF-8 to SIF

The ZIF-8 suspension (16 mL, absorbance: ~1 at 210 nm) was transferred to a 20 mL scintillation vial. Then, a methanolic solution of 1 M 2-mercaptomidazole (162 μL for SIF_{10mM}, 1.78 mL for SIF_{100mM}, and 4.00 mL for SIF_{200mM}) was added to the vial and mixed thoroughly. The mixture was left undisturbed for 63 h. The product solution was transferred into a 50 mL conical tube and washed twice, following the same treatment process described in Section 2.1.

To explore the effects of solvents other than MeOH, the ZIF-8 suspension (16 mL, absorbance: ~1 at 210 nm) was transferred into a 50 mL conical tube and centrifuged at 9000 rpm for 30 min. The supernatant was thoroughly discarded, and a different solvent such as THF for SIF_{THF}, ACE for SIF_{ACE}, IPA for SIF_{IPA}, EtOH for SIF_{EtOH}, or MeOH for SIF_{MeOH} was added. This process was repeated to ensure complete solvent exchange. Then, a methanolic solution of 250 mM 2-mercaptomidazole (4.00 mL) was added to the vial and mixed thoroughly. The mixture was left undisturbed for 63 h. The product solution was transferred into a 50 mL conical tube and washed twice, following the same treatment process described in Section 2.1.

To prepare SIF_{imi}, the ZIF-8 suspension (400 mL, absorbance: ~1 at 210 nm) was transferred into 50 mL conical tubes and centrifuged at 9000 rpm for 30 min. The supernatant was thoroughly discarded, and a methanolic solution of 125 mM 2-methylimidazole (400 mL) was added. It was transferred to a 500 mL Erlenmeyer flask. Then, a methanolic solution of 1 M 2-mercaptomidazole (100 mL) was added to the Erlenmeyer flask and mixed thoroughly. The mixture was left undisturbed for 63 h. The product solution was transferred into 50 mL conical tubes and washed twice, following the same treatment process described in Section 2.1.

All ZIF-8 and SIF materials were dried under vacuum overnight at 60°C for further characterization, if needed.

Section 3. Materials characterization

A FEI Tecnai TF30ST transmission electron microscope with a ZrO/W(100) Schottky emitter at 300 kV and a TESCAN S8000 scanning electron microscope in the Core-Facility Center for Photochemistry & Nanomaterials, Gyeongsang National University were used for morphological characterization. All samples were diluted 11-fold in methanol for TEM measurement. A 10 μL aliquot of the suspension was drop-cast onto a 400-mesh copper TEM grid and allowed to dry under ambient conditions. A JEM-ARM200F Cs-corrected scanning transmission electron

microscope (JEOL) in the High-Tech Materials Analysis Core Facility was used for elemental line mapping. Powder X-ray diffraction was performed using a D8 Advanced A25 (Bruker). Infrared spectra were obtained using a Nicolet iS5 FTIR spectrometer (Thermo Fisher Scientific) equipped with an iD7 ATR accessory. A 300 MHz FT-NMR spectrometer (AVANCE III 300, Bruker) was used to determine the ligand exchange ratio. All ZIF-8 and SIF materials were digested in a 9:1 (v/v) mixture of D_2O and D_2SO_4 using 10 mg of each sample. ^{13}C solid-state NMR was conducted using an AvanceNeo500 (Bruker). A Belsorp-mini II (MicrotracBEL) was used to analyze the Brunauer-Emmett-Teller (BET) surface area by measuring N_2 physisorption at 77 K. All dried powder was pretreated at 150°C for 6 h before BET measurement under vacuum. To determine the amount of adsorbed Hg, inductively coupled plasma optical emission spectrometry (ICP-OES) was performed using a Thermo Fisher Scientific iCAP PRO XP Duo. X-ray photoelectron spectroscopy (XPS) was conducted to determine chemical binding composition using a Thermo Fisher Scientific (NEXSA G2).

Section 4. Image analysis

Curvature distribution and color mapping were performed using ImageJ and a custom MATLAB code.[2] A TEM image of a ZIF-8 or SIF material was preprocessed in ImageJ to generate a 2D mask image of individual particles by applying thresholding and Gaussian blur (σ = 2.0 pixels). The MATLAB code identified the mask image boundary and fitted a circle to the boundary. The inverse radius of the fitted circle corresponded to curvature, and the maximum curvature range was determined. Mapping colors were assigned based on curvature ranges. The MATLAB code is provided below.

4.1. Curvature.m

```
scale_bar=100/569.5;%nm/pixel of TEM image

A = 'Filename.tif';
[filepath, name, ext] = fileparts(A);
outputFileName = strcat(name, '_curv', ext);
outputFilePath = fullfile(filepath, outputFileName);

mask=imread(A);
I0=imread(A);

boundary_all = bwboundaries(mask');

figure(1)
imshow(I0)
hold on
```

4.3. calCurv.m

```
function curv = calcCurv(x,y,n_points)
% PURPOSE: calculate curvature of a segement based on fitting a circle.
%   circfit cannot fit the circle well.
% HISTORY: modified by zihao, 20161010, chaged the circle fitting
curv = struct;
curv.radius = zeros(size(x));
curv.x = zeros(size(x));curv.y = zeros(size(x));
for count = 1:length(x)
    list_temp = initialList(count,length(x),n_points);
    x_temp = x(list_temp);  y_temp = y(list_temp);
    [curv.x(count),curv.y(count),curv.radius(count)] = CircleFitByPratt([x_temp,y_temp]);
    total_points = length(x_temp); index_mid = round((1+total_points)/2);
    x1 = x_temp(1);    x2 = x_temp(index_mid);    x3 = x_temp(total_points);
    y1 = y_temp(1);    y2 = y_temp(index_mid);    y3 = y_temp(total_points);
    vec_1 = [x2-x1,y2-y1,0];    vec_2 = [x3-x2,y3-y2,0];
```

```
for j = 1: length(boundary_all)
boundary=boundary_all{j};

points_fitting=68;
half_points=floor(points_fitting/2);

for i = 1 : length(boundary)
    %figure(1);clf;
    %imshow(I0)
    %hold on
    %plot(boundary(:,1),boundary(:,2),'LineWidth',3)


    if(i-half_points<1)
        fitting_list_1part=boundary(i-half_points+length(boundary):end,:);
        fitting_list_2part=boundary(i:i+half_points,:);
        fitting_list = cat (1,fitting_list_1part,fitting_list_2part);
    elseif(i+half_points>length(boundary))
        fitting_list_1part=boundary(i-half_points:end,:);
        N_still_need=points_fitting-(length(boundary)-(i-half_points)+1);
        fitting_list_2part=boundary(1:N_still_need,:);
        fitting_list = cat (1,fitting_list_1part,fitting_list_2part);
    else
        fitting_list = boundary(i-half_points:i+half_points,:);
    end

     [xx,yy] = smoothCurve(boundary(:,1), boundary(:,2), points_fitting);
     curv = calcCurv(xx, yy, points_fitting);

    %viscircles([x,y],R);
    %drawnow

end
 curvs=curv.curvature;

cb = jet(100);
curvs=abs(curvs / scale_bar);

min_curv=0;
max_curv=0.12;

colors=[];
for i = 1:length(boundary)
    curvA=curvs(i);
    if(isnan(curvA))
       curvA=0;
    end
    if(isinf(curvA))
        curvA=0;
    end
    index = floor(99*(curvA-min_curv)/max_curv+1);
    if(index>100)
        index=100;
    elseif(index<1)
        index=1;
    end
    color = cb (index,:) ;
    colors = cat(1,colors,color);
end
```

```
    cross_12 = cross(vec_2,vec_1);
    curv.radius(count) = curv.radius(count) * sign(cross_12(3));
end
curv.curvature = 1./curv.radius;
end
function list_temp = initialList(index,maximum,n_points)
list_temp = index - floor(n_points/2) : index + floor(n_points/2);
for count = 1 : length(list_temp)
    if list_temp(count) <= 0
        list_temp(count) = list_temp(count) + maximum;
    elseif list_temp(count) > maximum
        list_temp(count) = list_temp(count) - maximum;
    end
end
end
%% fitting functions
function [x,y,R] = CircleFitByPratt(XY)
% https://www.mathworks.com/matlabcentral/fileexchange/22643-circle-fit--pratt-method-
%--------------------------------------------------------------------------
%
%     Circle fit by Pratt
%      V. Pratt, "Direct least-squares fitting of algebraic surfaces",
%      Computer Graphics, Vol. 21, pages 145-152 (1987)
%
%     Input:  XY(n,2) is the array of coordinates of n points x(i)=XY(i,1), y(i)=XY(i,2)
%
%     Output: Par = [a b R] is the fitting circle:
%                           center (a,b) and radius R
%
%     Note: this fit does not use built-in matrix functions (except "mean"),
%           so it can be easily programmed in any programming language
%
%--------------------------------------------------------------------------

n = size(XY,1);      % number of data points

centroid = mean(XY);   % the centroid of the data set

%     computing moments (note: all moments will be normed, i.e. divided by n)

Mxx=0; Myy=0; Mxy=0; Mxz=0; Myz=0; Mzz=0;

for i=1:n
    Xi = XY(i,1) - centroid(1);  %  centering data
    Yi = XY(i,2) - centroid(2);  %  centering data
    Zi = Xi*Xi + Yi*Yi;
    Mxy = Mxy + Xi*Yi;Mxx = Mxx + Xi*Xi;Myy = Myy + Yi*Yi;
    Mxz = Mxz + Xi*Zi;Myz = Myz + Yi*Zi;Mzz = Mzz + Zi*Zi;
end
Mxx = Mxx/n;Myy = Myy/n;Mxy = Mxy/n;
Mxz = Mxz/n;Myz = Myz/n;Mzz = Mzz/n;
%    computing the coefficients of the characteristic polynomial

Mz = Mxx + Myy;
Cov_xy = Mxx*Myy - Mxy*Mxy;
Mxz2 = Mxz*Mxz;
Myz2 = Myz*Myz;

A2 = 4*Cov_xy - 3*Mz*Mz - Mzz;
A1 = Mzz*Mz + 4*Cov_xy*Mz - Mxz2 - Myz2 - Mz*Mz*Mz;
A0 = Mxz2*Myy + Myz2*Mxx - Mzz*Cov_xy - 2*Mxz*Myz*Mxy + Mz*Mz*Cov_xy;
A22 = A2 + A2;
```

```
boundary_plot = plot(boundary(:,1),boundary(:,2),'LineWidth',3,'color','b');
pause(0.1);
set(boundary_plot.Edge,
'ColorBinding','interpolated','ColorData',uint8(255*[colors,ones(length(colors),1)]'));
set(gca,'position',[0 0 1 1],'units','normalized');
set(figure(1),'Position', [0 0 512 512]);

disp(max(curvA))

end

saveas(figure(1),outputFilePath)
%%show the color bar%%
figure(2)
colormap(jet);
caxis([min_curv,max_curv]);
foo=colorbar();

set(gca,'position',[-0.5 0.1 0.8 0.8],'units','normalized');
set(figure(2),'Position', [0 0 200 512]);
set(gca,'visible','off')
saveas(figure(2),'colorbar.tif')
```

4.2. SmoothCurve.m

```
function [xx,yy] = smoothCurve(x,y,n_points)
% smooth the edge curve
xx = zeros(size(x));    yy = zeros(size(y));
for count = 1:length(x)
    list_temp = initialList(count,length(x),n_points);
    x_temp = x(list_temp);  y_temp = y(list_temp);
    xx(count) = mean(x_temp);
    yy(count) = mean(y_temp);
end
end
function list_temp = initialList(index,maximum,n_points)
list_temp = index - floor(n_points/2) : index + floor(n_points/2);
for count = 1 : length(list_temp)
    if list_temp(count) <= 0
        list_temp(count) = list_temp(count) + maximum;
    elseif list_temp(count) > maximum
        list_temp(count) = list_temp(count) - maximum;
    end
end
end
```

```
epsilon=1e-12;
ynew=1e+20;
IterMax=20;
xnew = 0;

%    Newton's method starting at x=0

for iter=1:IterMax
    yold = ynew;
    ynew = A0 + xnew*(A1 + xnew*(A2 + 4.*xnew*xnew));
    if (abs(ynew)>abs(yold))
        disp('Newton-Pratt goes wrong direction: |ynew| > |yold|');
        xnew = 0;
        break;
    end
    Dy = A1 + xnew*(A22 + 16*xnew*xnew);
    xold = xnew;
    xnew = xold - ynew/Dy;
    if (abs((xnew-xold)/xnew) < epsilon), break, end
    if (iter >= IterMax)
        disp('Newton-Pratt will not converge');
        xnew = 0;
    end
    if (xnew<0.)
        fprintf(1,'Newton-Pratt negative root:  x=%f\n',xnew);
        xnew = 0;
    end
end

%    computing the circle parameters

DET = xnew*xnew - xnew*Mz + Cov_xy;
Center = [Mxz*(Myy-xnew)-Myz*Mxy , Myz*(Mxx-xnew)-Mxz*Mxy]/DET/2;

Par = [Center+centroid , sqrt(Center*Center'+Mz+2*xnew)];
x = Par(1);y = Par(2);R = Par(3); % added by zihao, 20161010
end    %    CircleFitByPratt
```

Section 5. Hg^{2+} ion adsorption

To determine the adsorption model and maximum adsorption capacity, 10 mg of ZIF-8, SIF_{imi}, and $SIF_{200\ mM}$ were each transferred into a 20 mL scintillation vial. Then, $HgCl_2$ aqueous solutions at varying concentrations (0.5, 1, 2, 4, 6, 8, and 10 mM) were added to each sample. The mixtures were stirred at 300 rpm in a 25°C water bath for 12 h. After incubation, the mixtures were centrifuged at 8000 rpm for 10 min, and 5 mL of the supernatant was extracted. The supernatant

was then diluted 42-fold using 12% nitric acid to measure the mercury concentration via ICP-OES. The adsorption capacity was calculated using the following equation.

$$q_e = \frac{(C_0 - C_e) \times V}{m}$$

q_e = Adsorption capacity

C_0 = Initial Hg^{2+} ion concentration (mg/L)

C_e = Equilibrated Hg^{2+} ion concentration after adsorption (mg/L)

V = Solution volume (L)

m = Weight of the adsorbent (g)

The adsorption-desorption cycle test was performed using a modified method.[3] A total of 20 mg of ZIF-8, SIF_{imi}, and SIF_{200mM} were each transferred into a 20 mL scintillation vial, followed by the addition of 10 mL of a 7.2 mM $HgCl_2$ aqueous solution (1444 ppm). The suspensions were stirred at 300 rpm for 2 h in a 25°C water bath. After adsorption, the suspensions were centrifuged at 9000 rpm for 10 min, and the supernatant was extracted for ICP-OES analysis. The solid residues were washed with DI water, collected again by centrifugation, and then dispersed in a 1 mM HCl (pH 3) solution containing 1 wt% thiourea solution for 2 h. After desorption, the adsorbents were collected, washed twice with methanol, dried under active vacuum at 60°C for 1 h, and then reused for the next adsorption cycle. This process was repeated four more times.

The low concentration of Hg^{2+} adsorption (10 ppm) was performed as follows. 15 mg of each adsorbent (ZIF-8, SIF_{imi}, and SIF_{200mM}) were added to 20 mL glass vials containing 8 mL DI water and 1 mL of a 100 ppm $HgCl_2$ stock solution. The pH was adjusted to 3, 7, or 11 by dropwise addition of HCl (1 mL, pH 2) or NaOH (1 mL, pOH 2), and the suspensions were stirred at 300 rpm for 2 h at room temperature. After centrifugation (9000 rpm, 15 min), approximately 5 mL of supernatant was collected, diluted five-fold with 12% HNO_3, and analyzed by ICP-OES as described above. The pH 7 exposed sample residue was collected for XPS analysis and vacuum-dried at 100 °C for 24 h. In addition, the adsorption-desorption cycle test was also performed. Each cycle used 25 mg of adsorbent in 10 mL of a 10 ppm $HgCl_2$ solution. Samples were vortexed for 30 s and allowed to stand for 15 min, repeated twice per cycle, then centrifuged (9000 rpm, 15 min). The supernatant (~3 mL) was withdrawn, diluted with 12% HNO_3 (1 mL supernatant in 4

mL acid), and measured by ICP-OES. The adsorbent was then re-suspended in fresh 10 ppm $HgCl_2$ solution for the next cycle without additional desorption process, for a total of five cycles.

The multi-ion adsorption test was conducted as follows. 15 mg of each adsorbent was mixed with 10 mL of a solution containing seven metal ions (Hg^{2+}, Pb^{2+}, Ni^{2+}, Co^{2+}, Fe^{3+}, Cr^{3+}, and Cu^{2+}) at 10 ppm each. The mixtures were stirred at 300 rpm for 2 h and centrifuged (9000 rpm, 15 min). Approximately 5 mL of supernatant was collected, diluted five-fold with 12% HNO_3, and analyzed by ICP-OES.

Figures

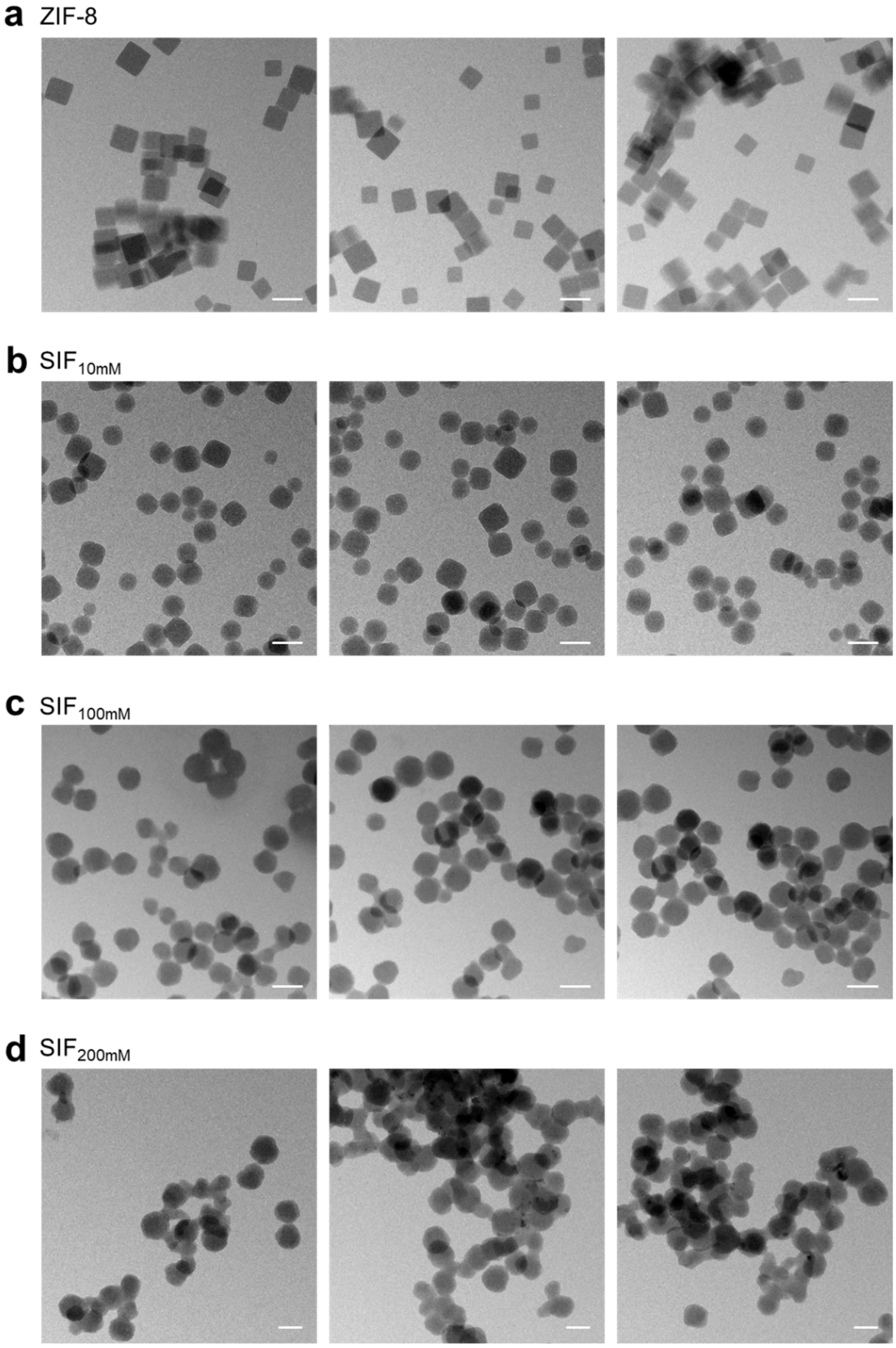

Figure S1. Representative TEM images of (a) ZIF-8, (b) SIF_{10mM}, (c) SIF_{100mM}, and (d) SIF_{200mM}. Scale bar: 100 nm.

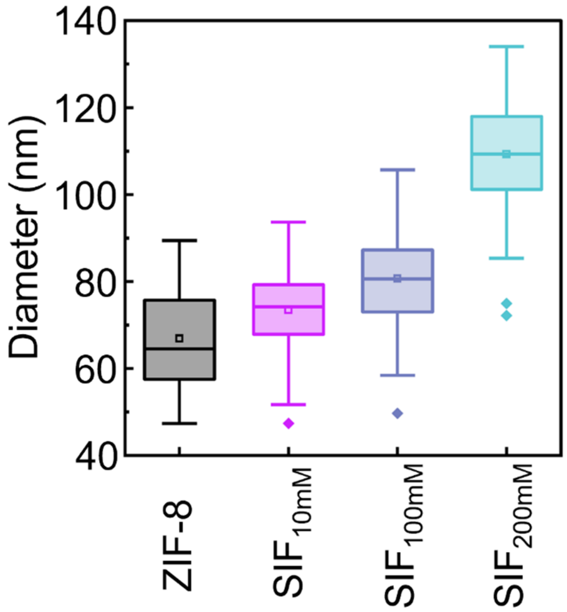

Figure S2. Box plots of the measured response in groups ZIF-8, SIF_{10mM}, SIF_{100mM}, and SIF_{200mM} following one-way ANOVA. Boxes span the interquartile range (25–75%), central lines denote medians, inset squares indicate means, whiskers extend to the most extreme values within 1.5 × interquartile range, and dots represent outliers.

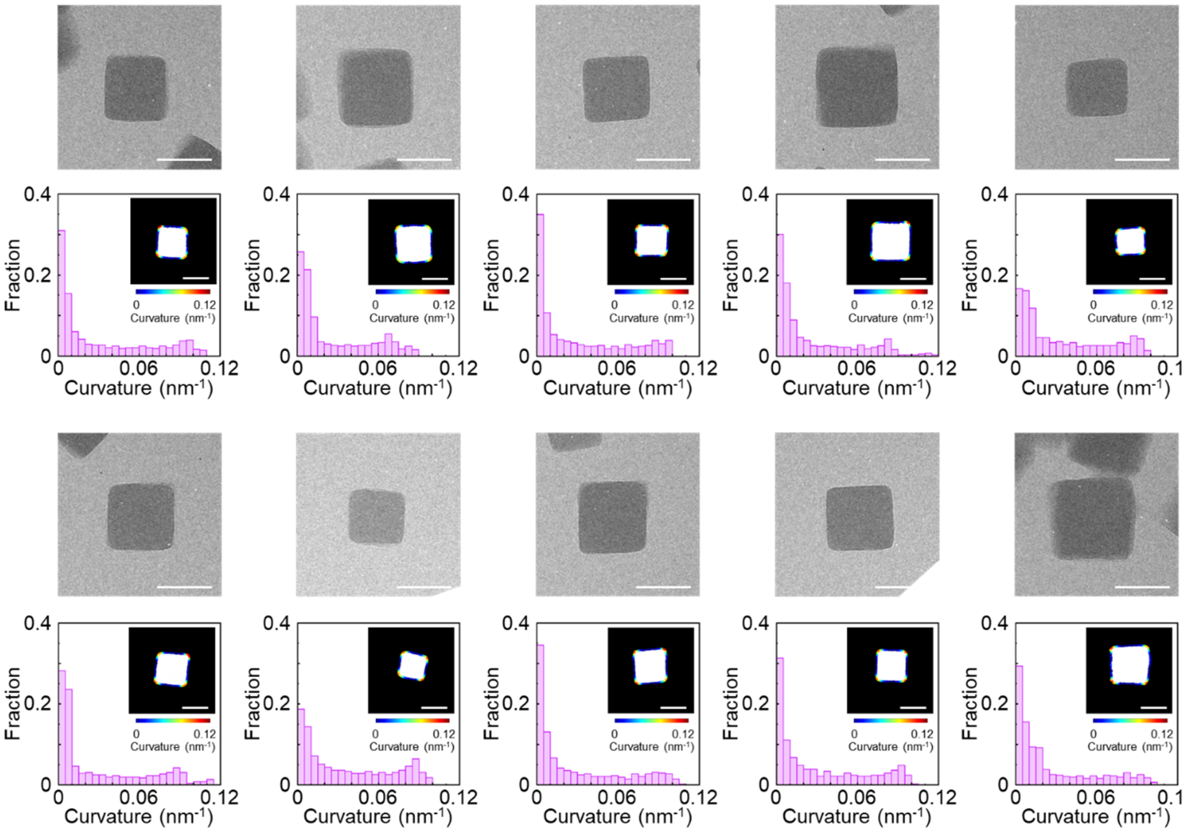

Figure S3. TEM images and curvature distribution of ten ZIF-8 particles. Scale bar: 50 nm.

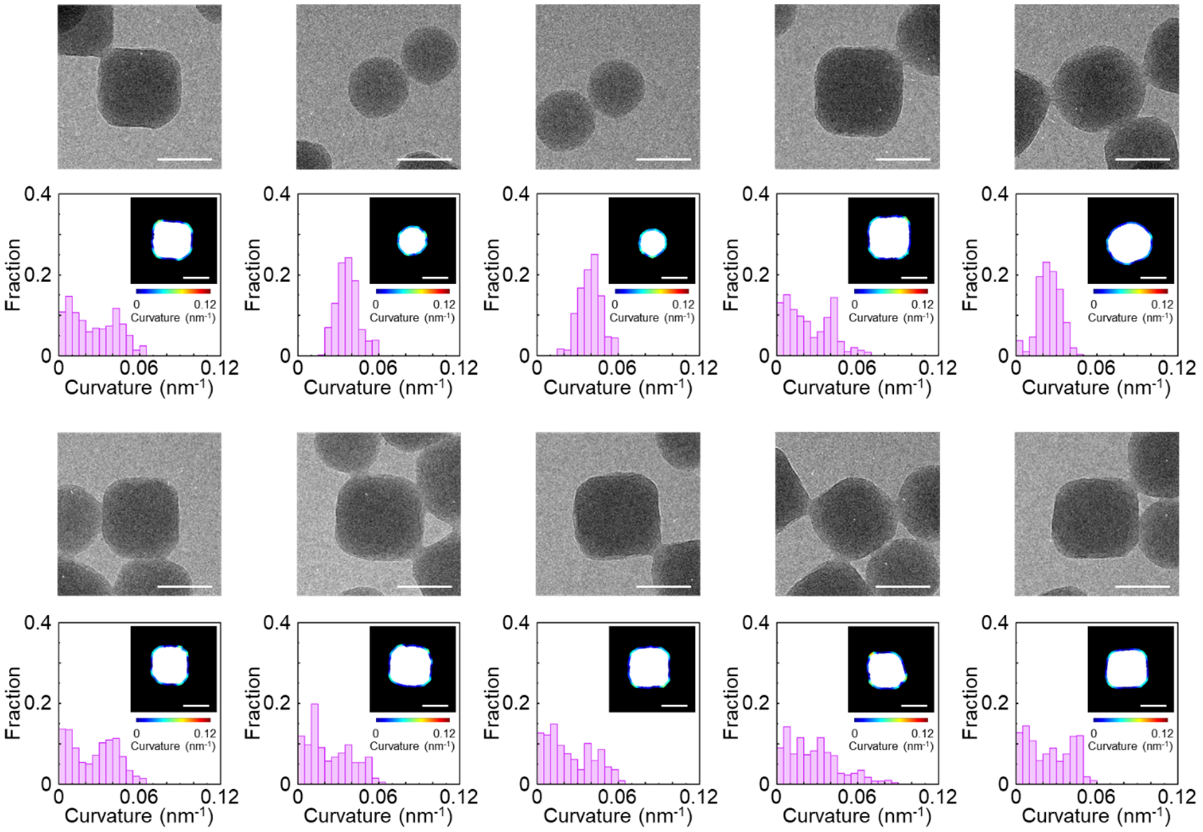

Figure S4. TEM images and curvature distribution of ten SIF_{10mM} particles. Scale bar: 50 nm.

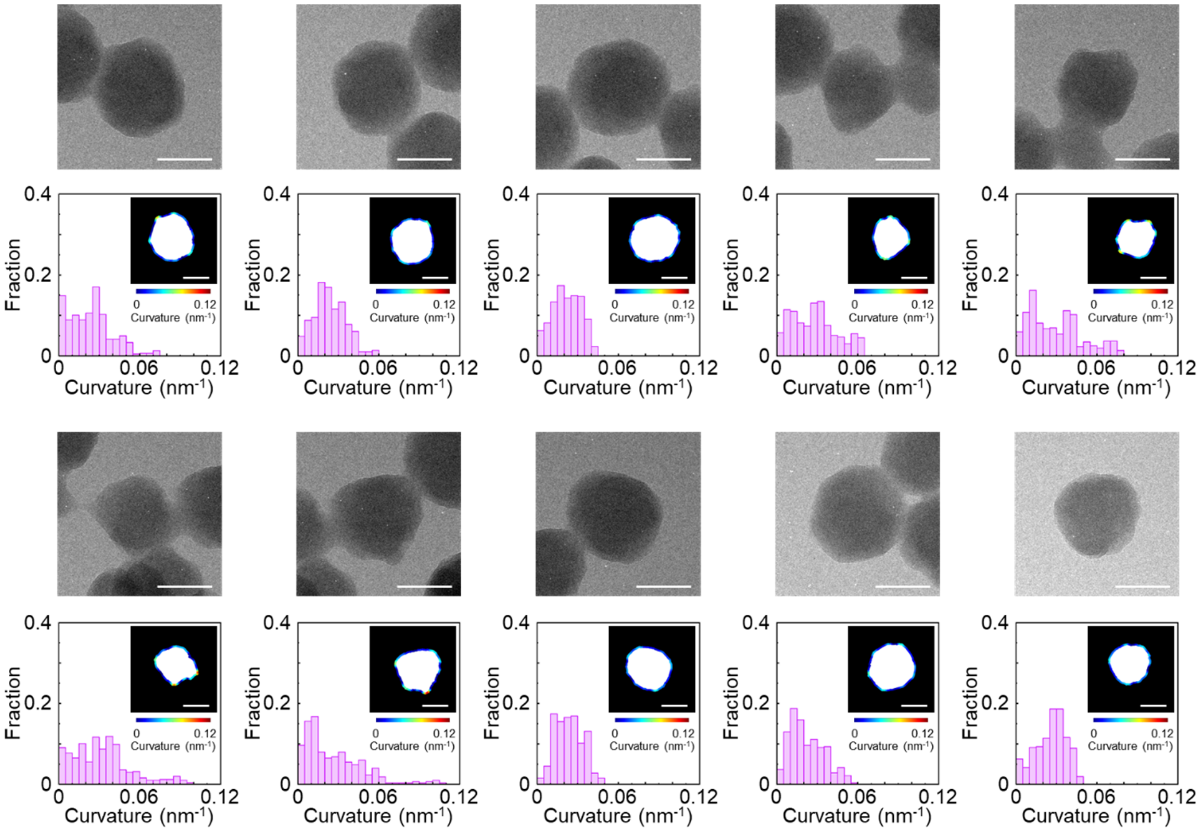

Figure S5. TEM images and curvature distribution of ten SIF_{100mM} particles. Scale bar: 50 nm.

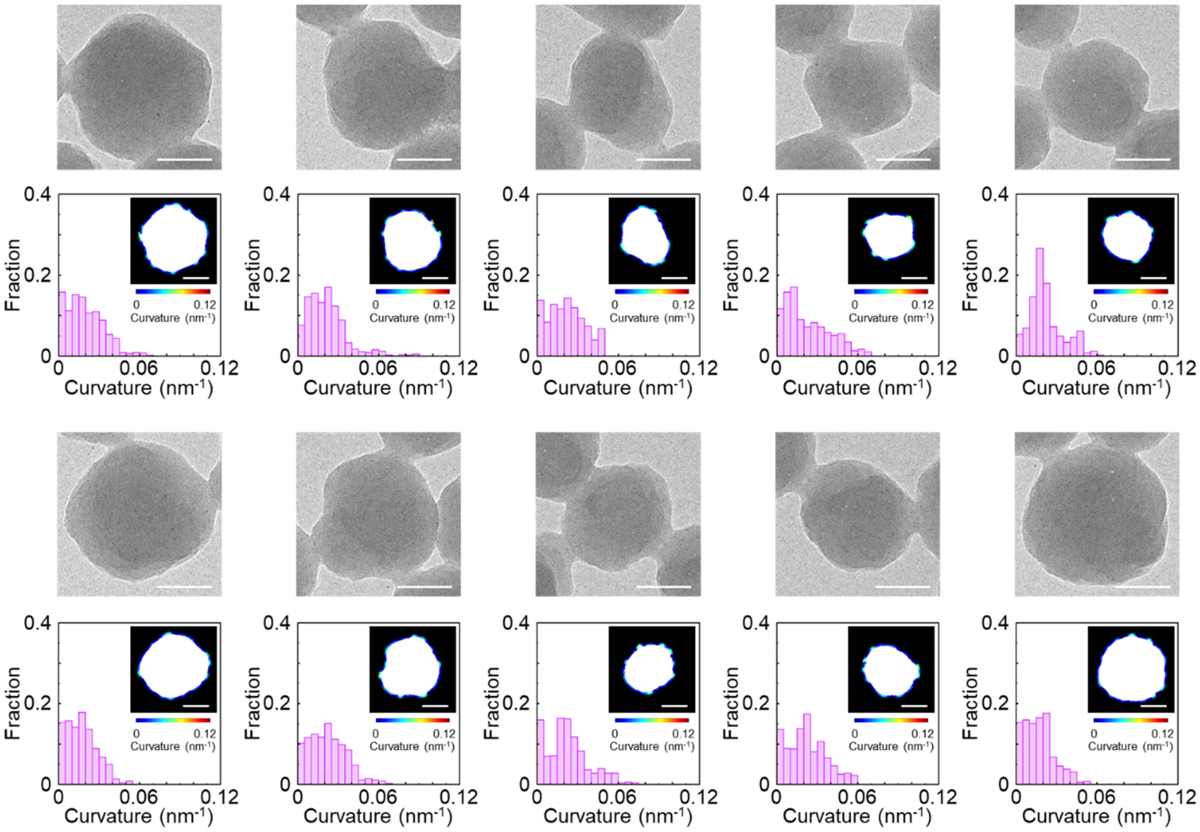

Figure S6. TEM images and curvature distribution of ten SIF_{200mM} particles. Scale bar: 50 nm.

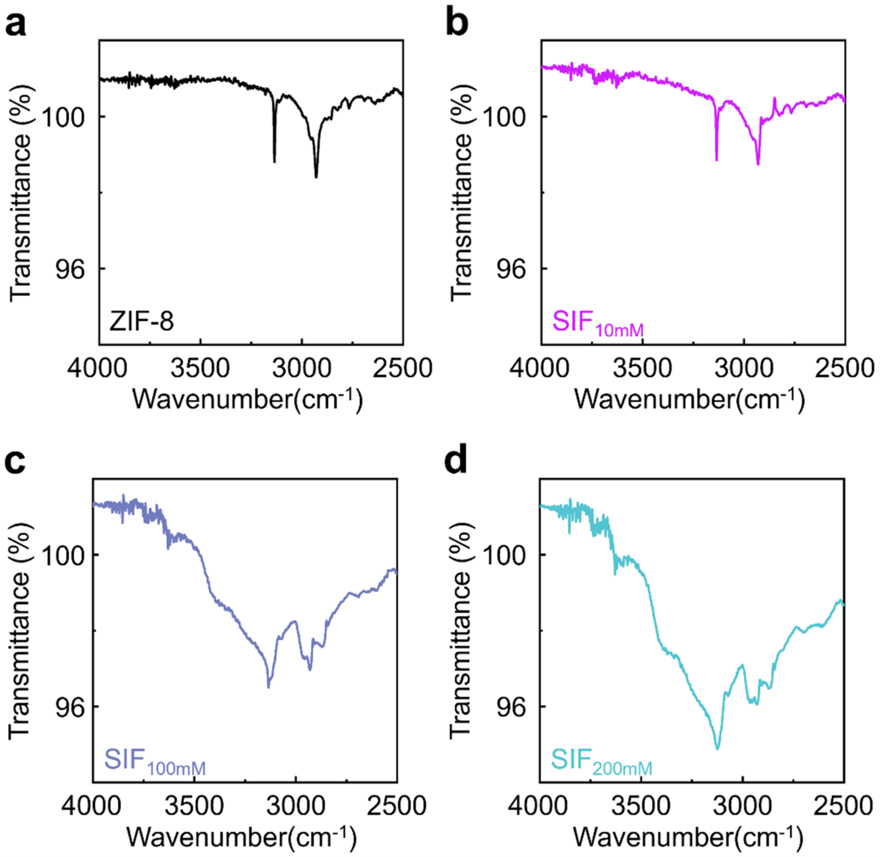

Figure S7. Magnified IR spectra of (a) ZIF-8, (b) SIF_{10mM}, (c) SIF_{100mM}, and (d) SIF_{200mM} in the 2500–4000 cm^{-1} region. C-H stretching bands at 2929 and 3135 cm^{-1} are clearly observed in ZIF-8 and SIF_{10mM}. In contrast, the bands in SIF_{100mM} and SIF_{200mM} are broadened and shifted, likely due to increased hydrogen bonding.

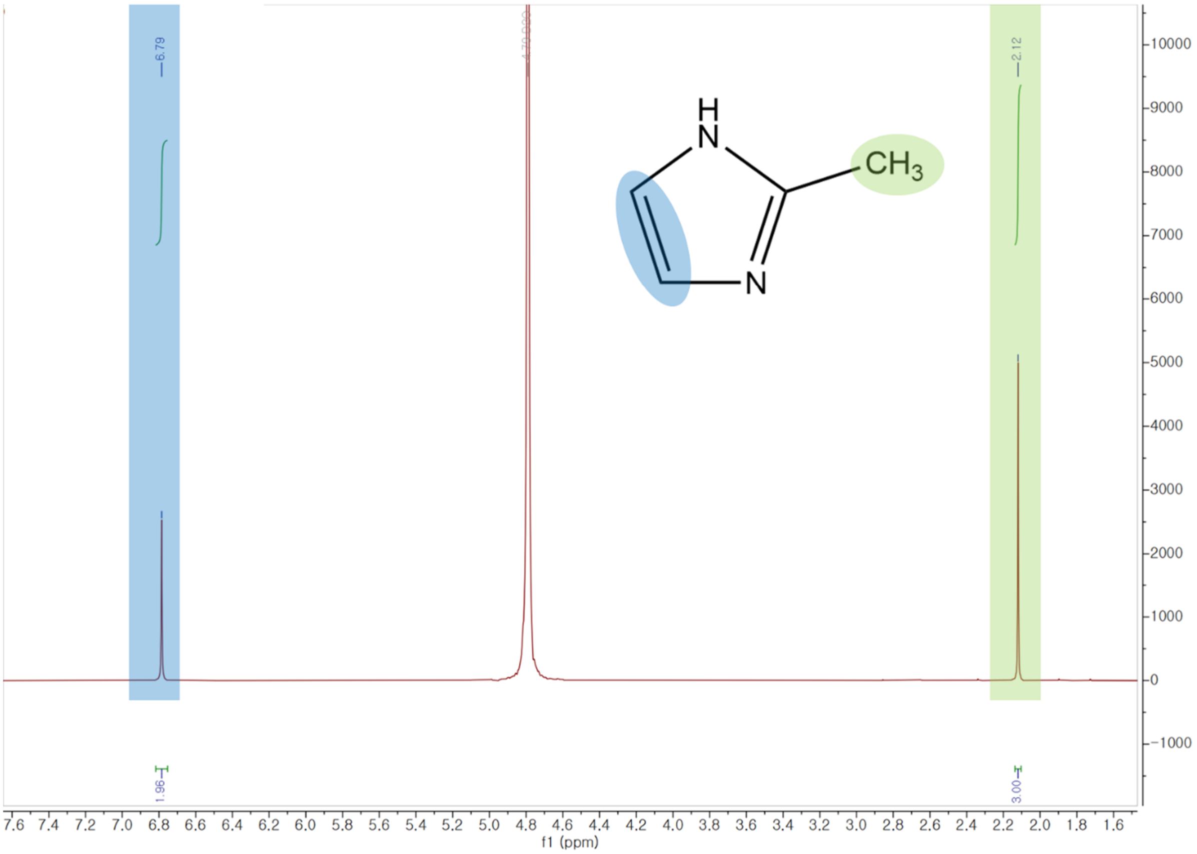

Figure S8. ^{1}H NMR spectrum of digested ZIF-8. The blue-highlighted peak at 6.8 ppm corresponds to the two protons in the imidazole ring (–CH=), while the green-highlighted peak at 2.1 ppm corresponds to the three protons in the methyl group ($–CH_3$).

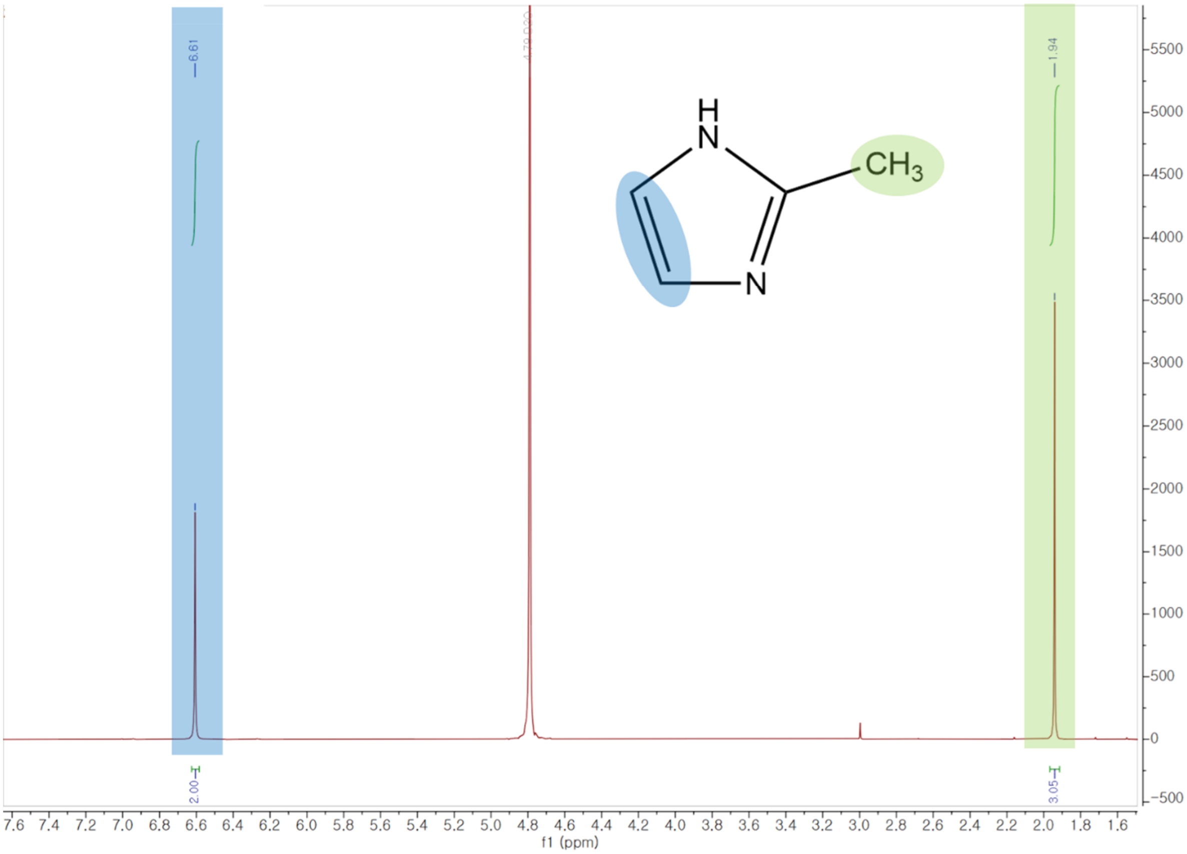

Figure S9. ^{1}H NMR spectrum of digested SIF_{10mM}. The blue-highlighted peak at 6.6 ppm corresponds to the two protons in the imidazole ring (–CH=), while the green-highlighted peak at 1.9 ppm corresponds to the three protons in the methyl group (–CH_3).

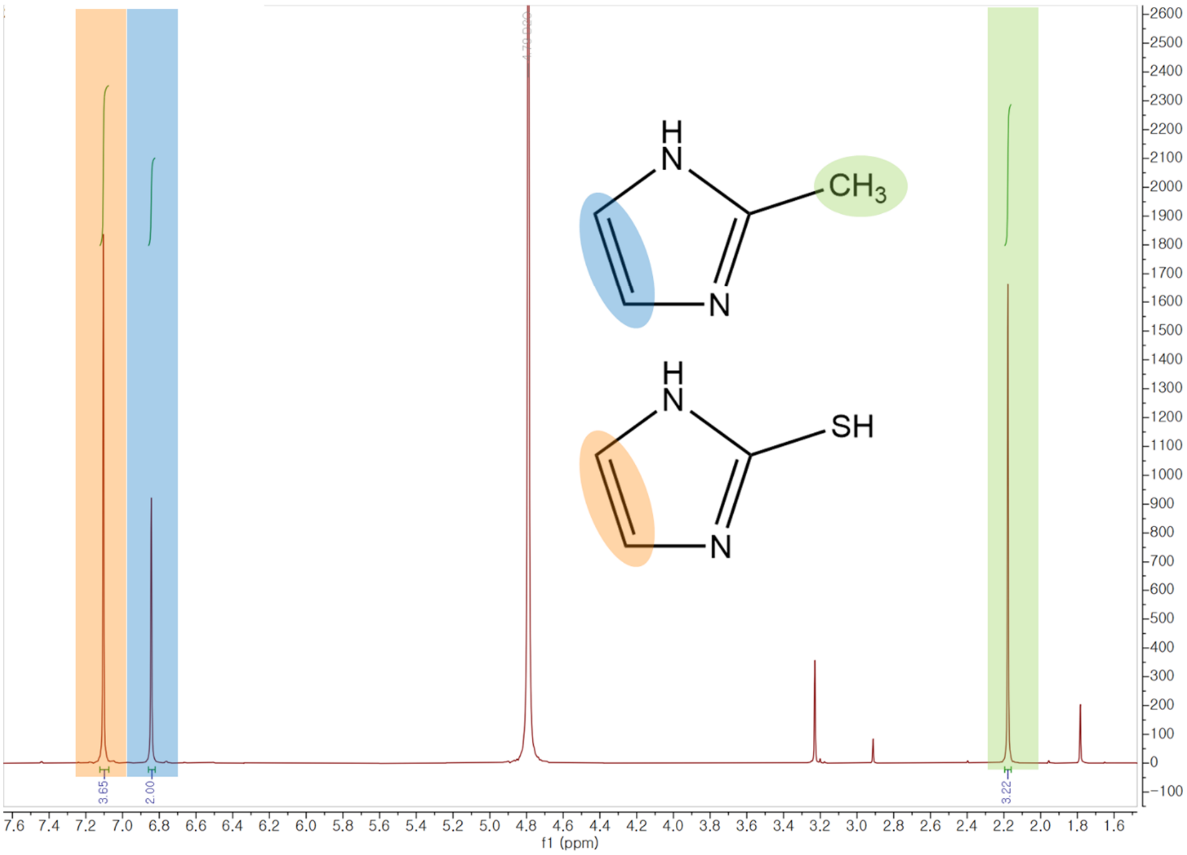

Figure S10. ^{1}H NMR spectrum of digested SIF_{100mM}. The light orange-highlighted peak at 7.1 ppm corresponds to the two protons in the mercaptoimidazole ring (–CH=). The blue-highlighted peak at 6.8 ppm corresponds to the two protons in the imidazole ring (–CH=), while the green-highlighted peak at 2.2 ppm corresponds to the three protons in the methyl group ($–CH_3$).

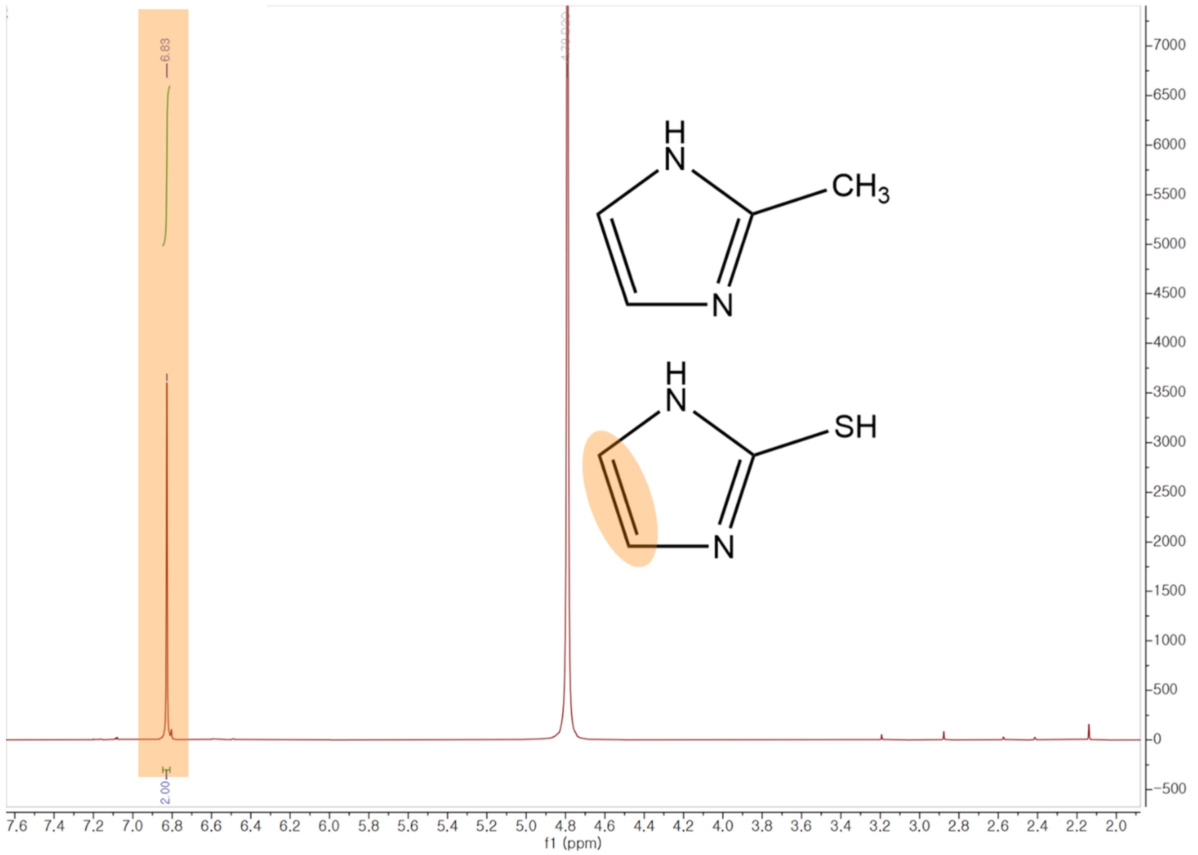

Figure S11. ^{1}H NMR spectrum of digested SIF$_{200mM}$. The light orange-highlighted peak at 6.8 ppm corresponds to the two protons in the mercaptoimidazole ring (–CH=).

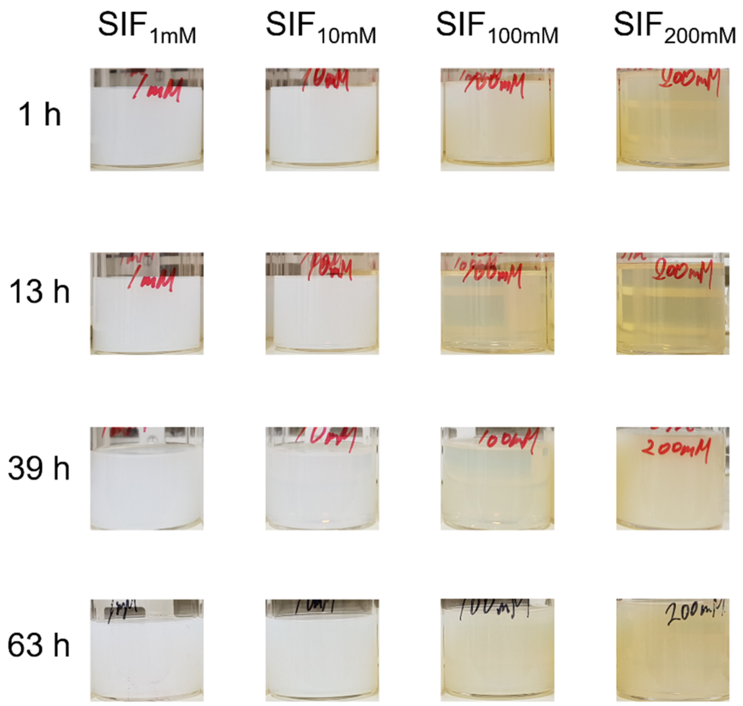

Figure S12. Digital photographs showing SIF_{1mM}, SIF_{10mM}, SIF_{100mM}, and SIF_{200mM} suspensions at 1, 13, 39, and 63 h, respectively. The darkness of the suspension illustrates the time-dependent changes occurring at different concentrations of 2-mercaptoimidazole.

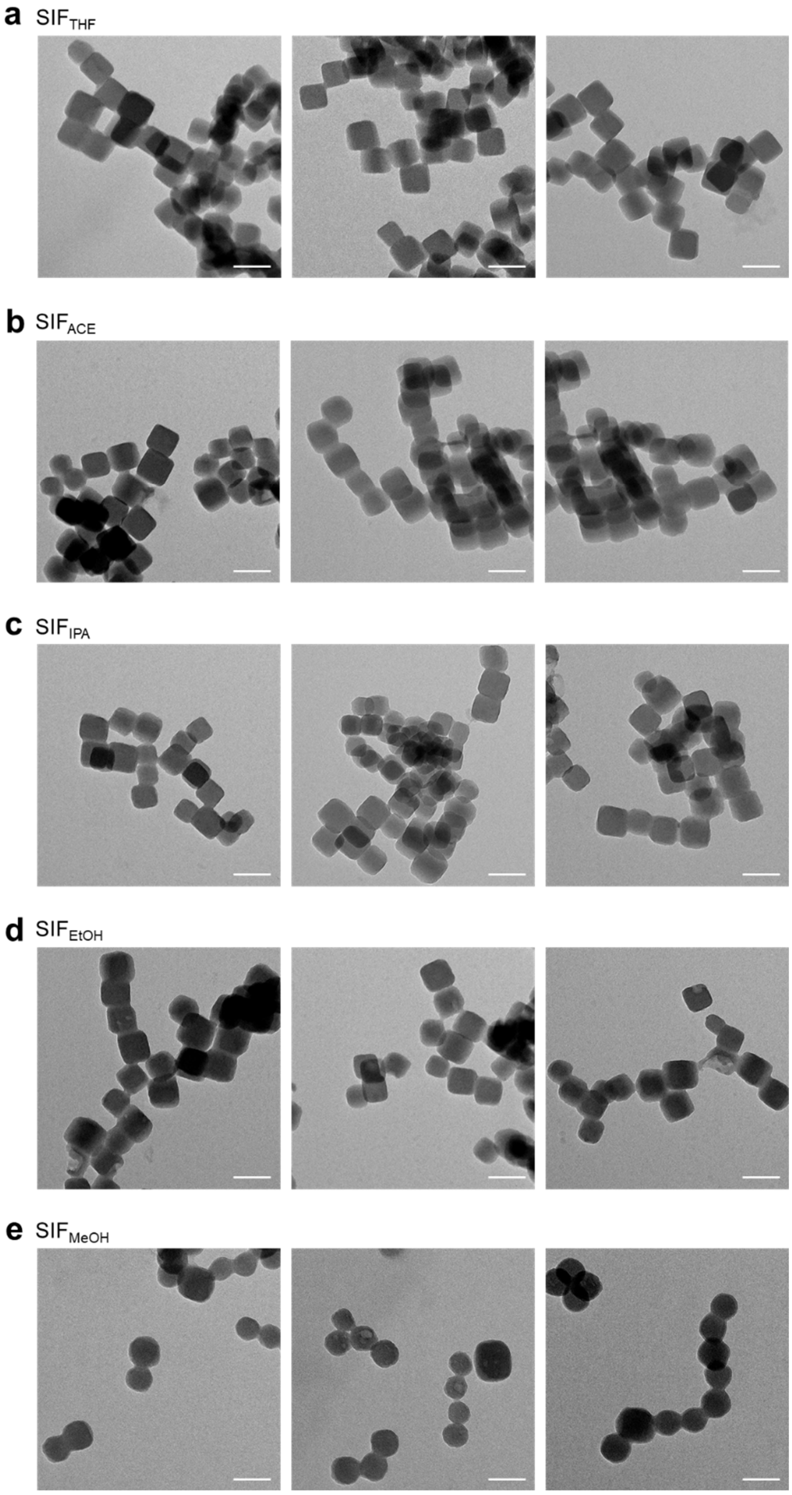

Figure S13. Representative TEM images of (a) SIF$_{THF}$, (b) SIF$_{ACE}$, (c) SIF$_{IPA}$, (d) SIF$_{EtOH}$, and (e) SIF$_{MeOH}$. Scale bar: 100 nm.

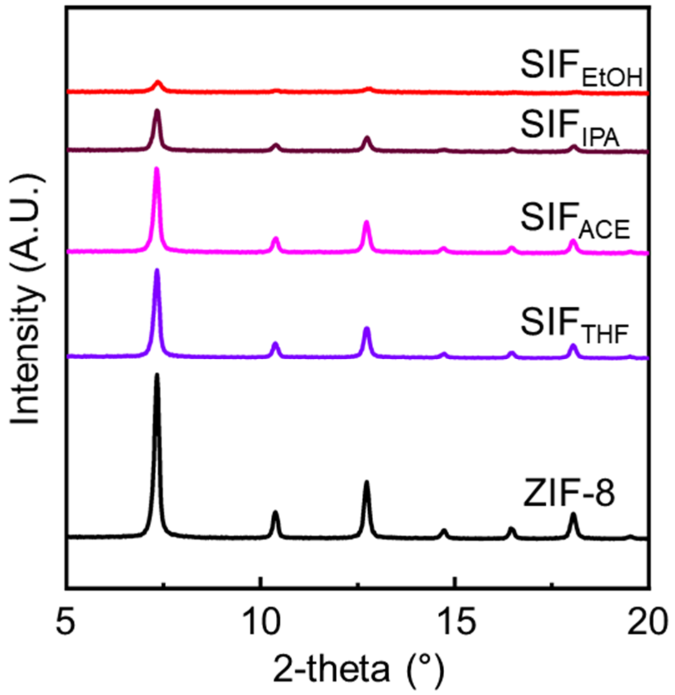

Figure S14. XRD patterns of ZIF-8 (black line), SIF_{THF} (purple line), SIF_{ACE} (magenta line), SIF_{IPA} (brown line), and SIF_{EtOH} (red line).

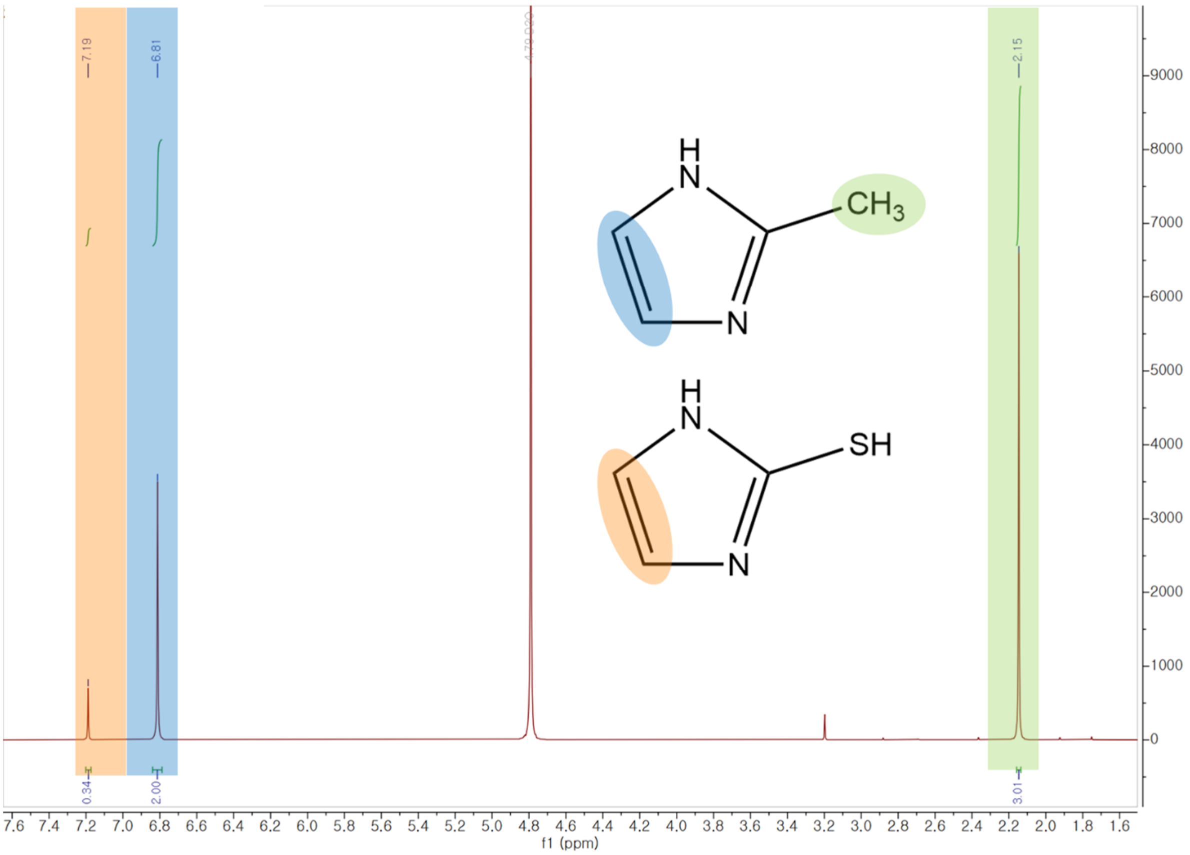

Figure S15. ^{1}H NMR spectrum of digested SIF_{THF}. The light orange-highlighted peak at 7.2 ppm corresponds to the two protons in the mercaptoimidazole ring (–CH=). The blue-highlighted peak at 6.8 ppm corresponds to the two protons in the imidazole ring (–CH=), while the green-highlighted peak at 2.1 ppm corresponds to the three protons in the methyl group (–CH_3).

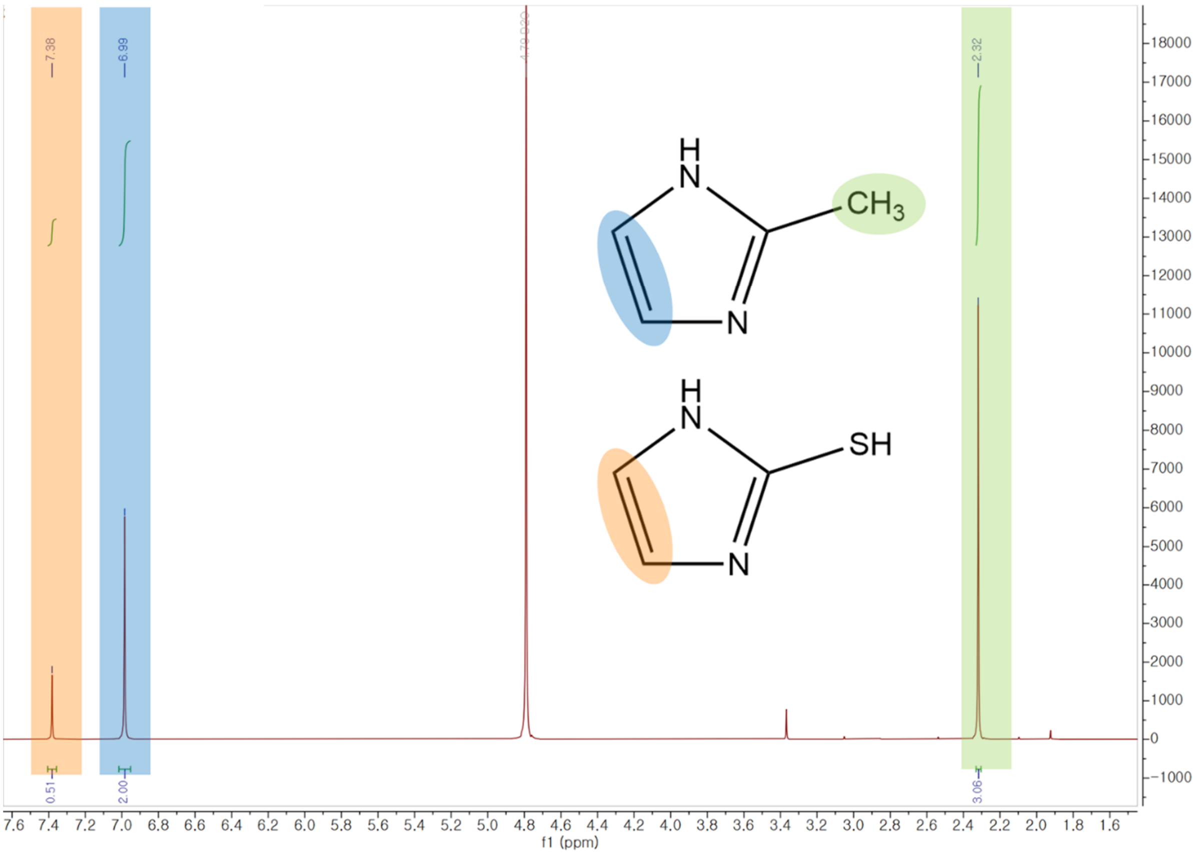

Figure S16. ^{1}H NMR spectrum of digested SIF$_{ACE}$. The light orange-highlighted peak at 7.4 ppm corresponds to the two protons in the mercaptoimidazole ring (–CH=). The blue-highlighted peak at 7.0 ppm corresponds to the two protons in the imidazole ring (–CH=), while the green-highlighted peak at 2.3 ppm corresponds to the three protons in the methyl group (–CH_3).

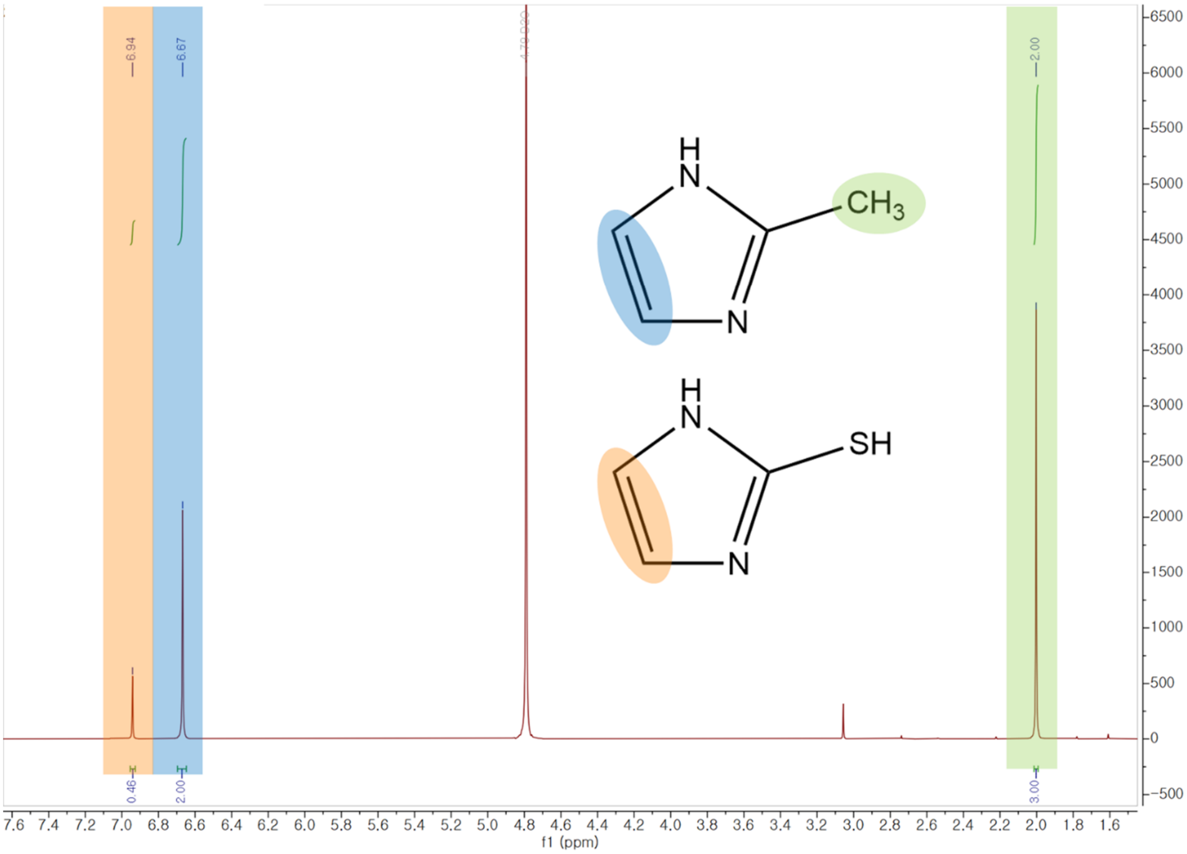

Figure S17. ^{1}H NMR spectrum of digested SIF$_{IPA}$. The light orange-highlighted peak at 6.9 ppm corresponds to the two protons in the mercaptoimidazole ring (–CH=). The blue-highlighted peak at 6.7 ppm corresponds to the two protons in the imidazole ring (–CH=), while the green-highlighted peak at 2.0 ppm corresponds to the three protons in the methyl group (–CH_3).

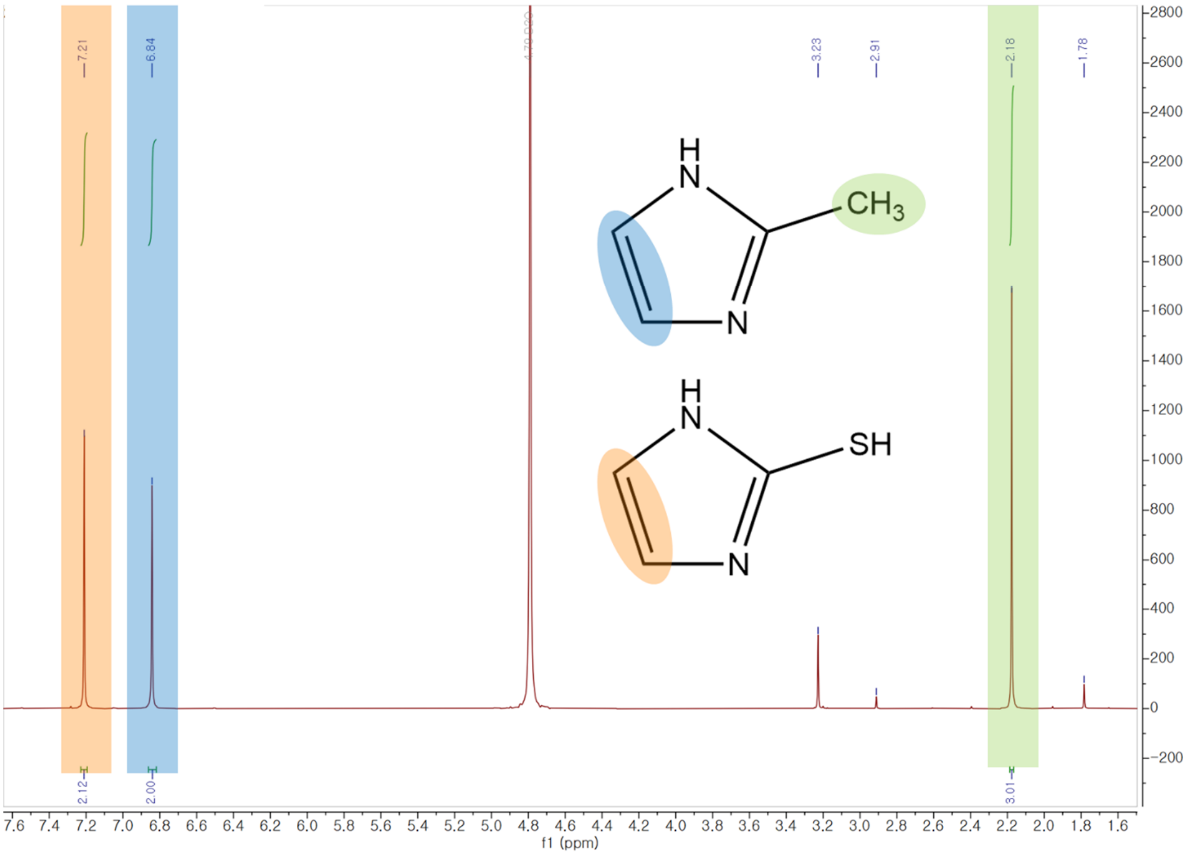

Figure S18. ^{1}H NMR spectrum of digested SIF_{EtOH}. The light orange-highlighted peak at 7.2 ppm corresponds to the two protons in the mercaptoimidazole ring (–CH=). The blue-highlighted peak at 6.8 ppm corresponds to the two protons in the imidazole ring (–CH=), while the green-highlighted peak at 2.2 ppm corresponds to the three protons in the methyl group ($–CH_3$).

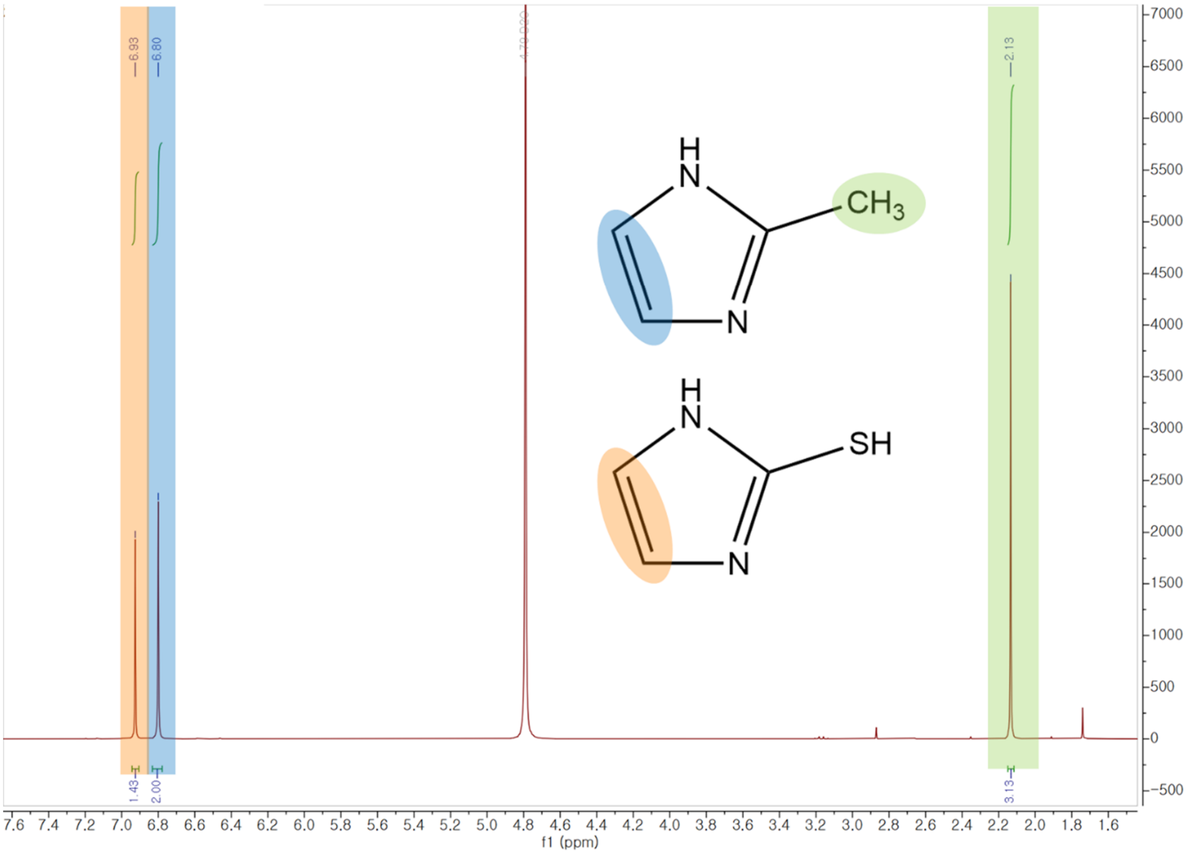

Figure S19. ^{1}H NMR spectrum of digested SIF_{MeOH}. The light orange-highlighted peak at 6.9 ppm corresponds to the two protons in the mercaptoimidazole ring (–CH=). The blue-highlighted peak at 6.8 ppm corresponds to the two protons in the imidazole ring (–CH=), while the green-highlighted peak at 2.1 ppm corresponds to the three protons in the methyl group ($–CH_3$).

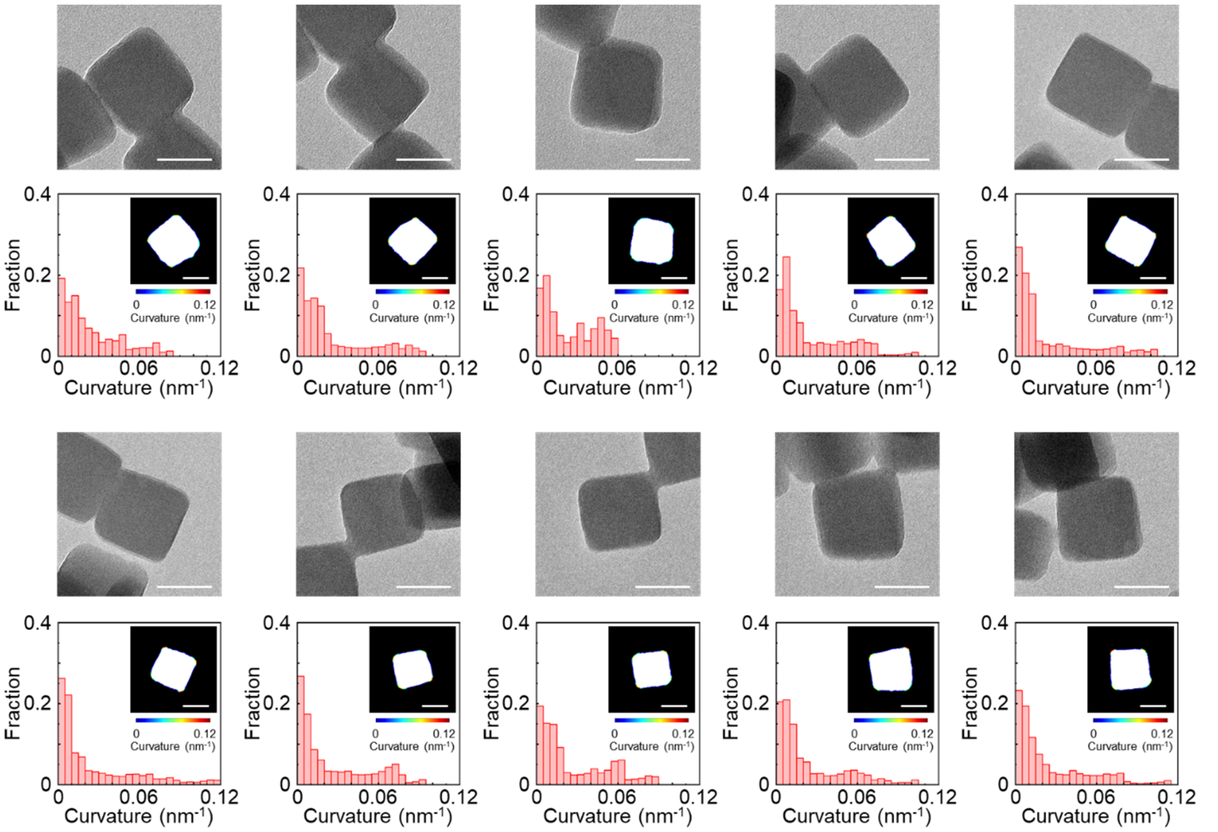

Figure S20. TEM images and curvature distribution of ten SIF_{THF} particles. Scale bar: 50 nm.

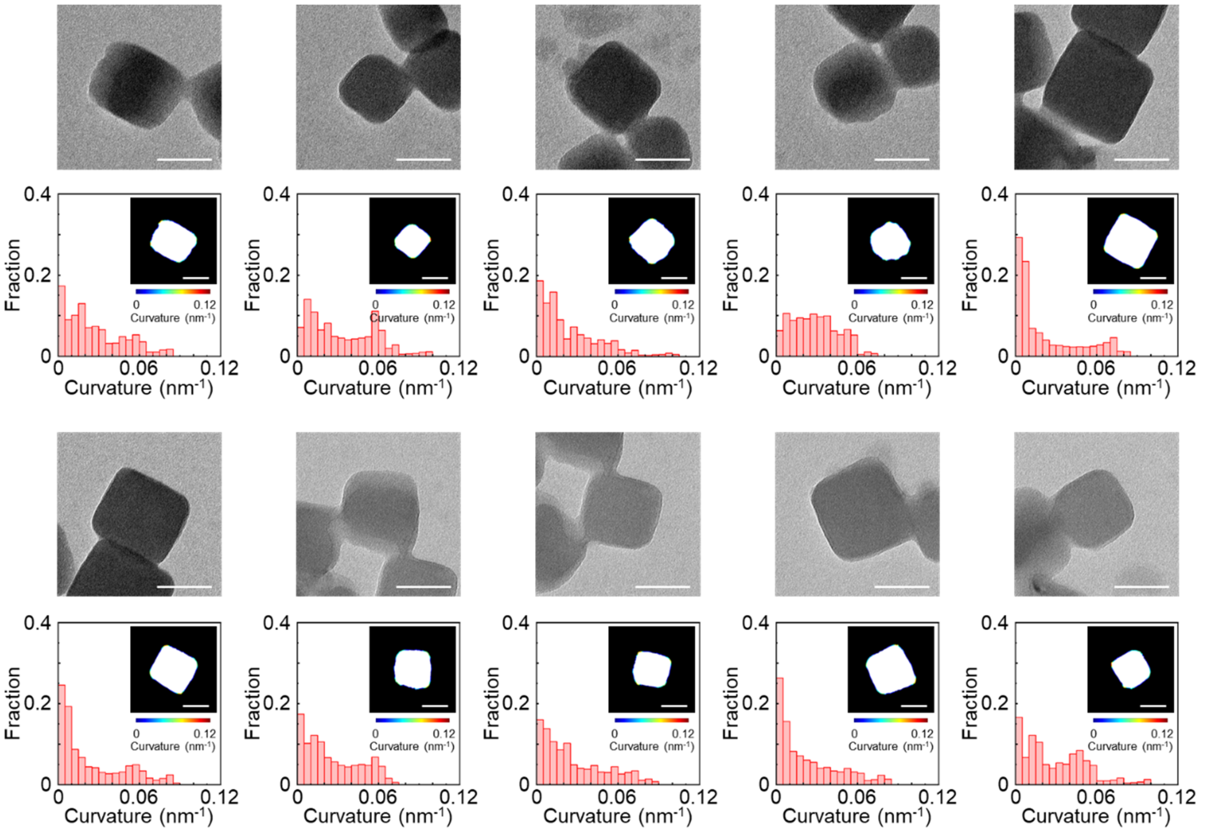

Figure S21. TEM images and curvature distribution of ten SIF_{ACE} particles. Scale bar: 50 nm.

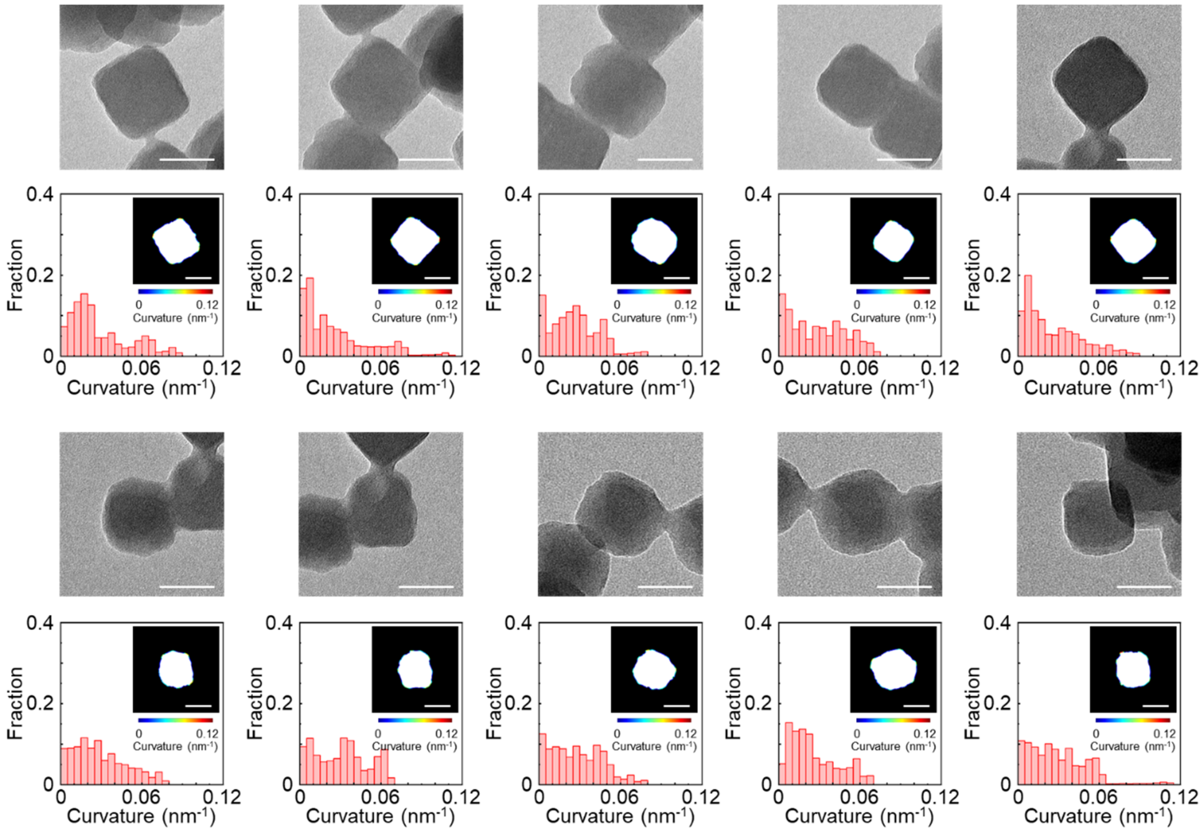

Figure S22. TEM images and curvature distribution of ten SIF_{IPA} particles. Scale bar: 50 nm.

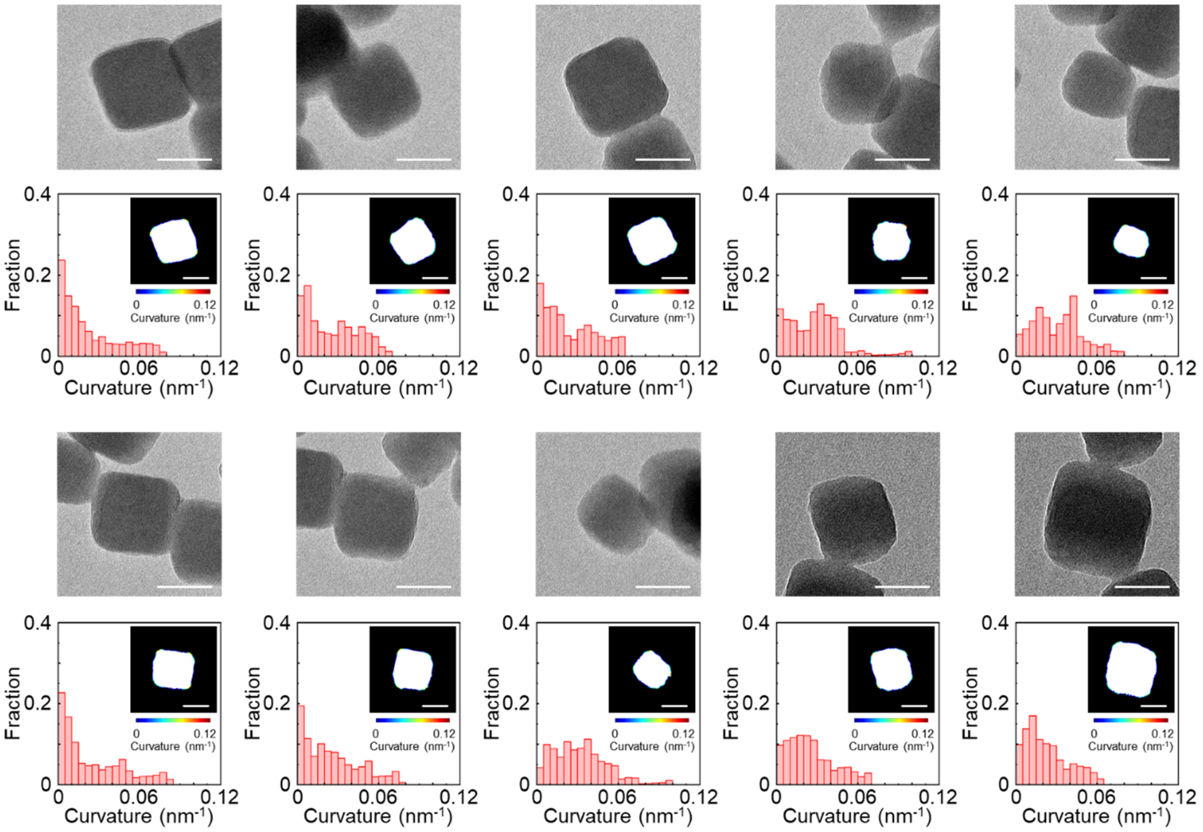

Figure S23. TEM images and curvature distribution of ten SIF_{EtOH} particles. Scale bar: 50 nm.

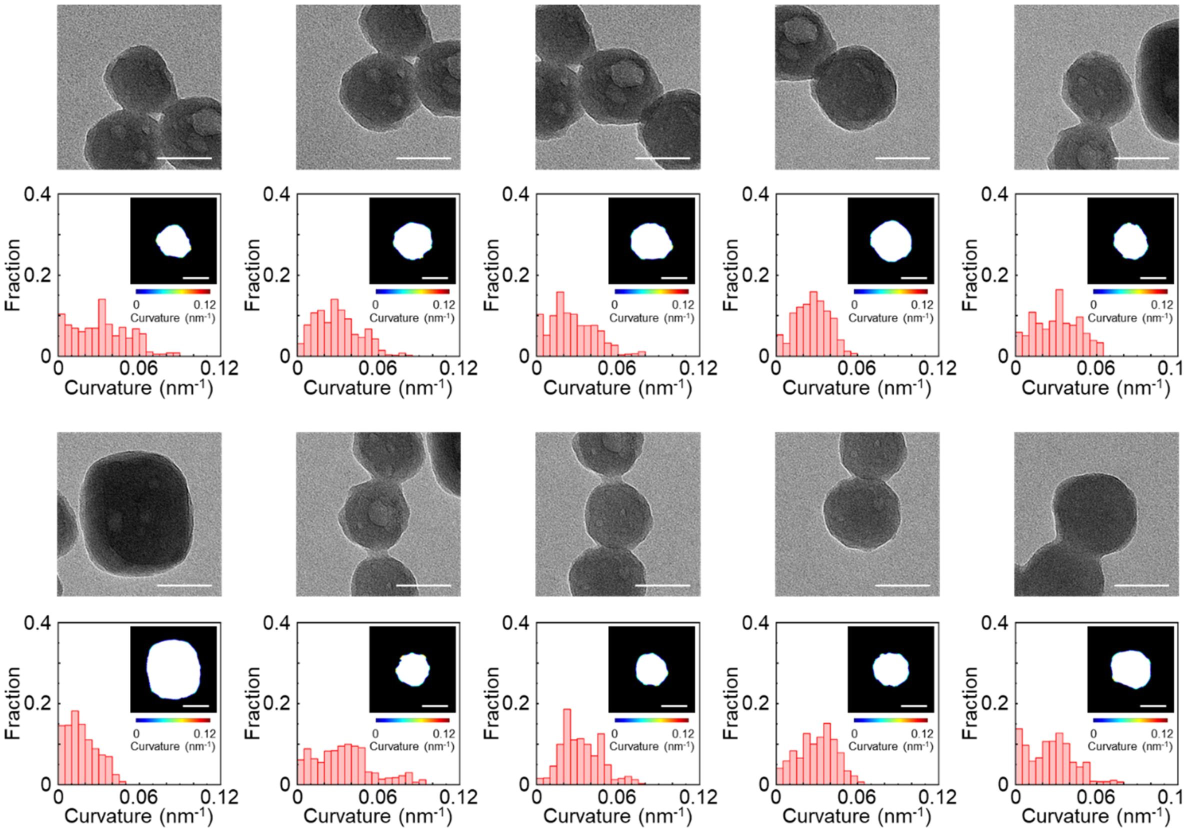

Figure S24. TEM images and curvature distribution of ten SIF_{MeOH} particles. Scale bar: 50 nm.

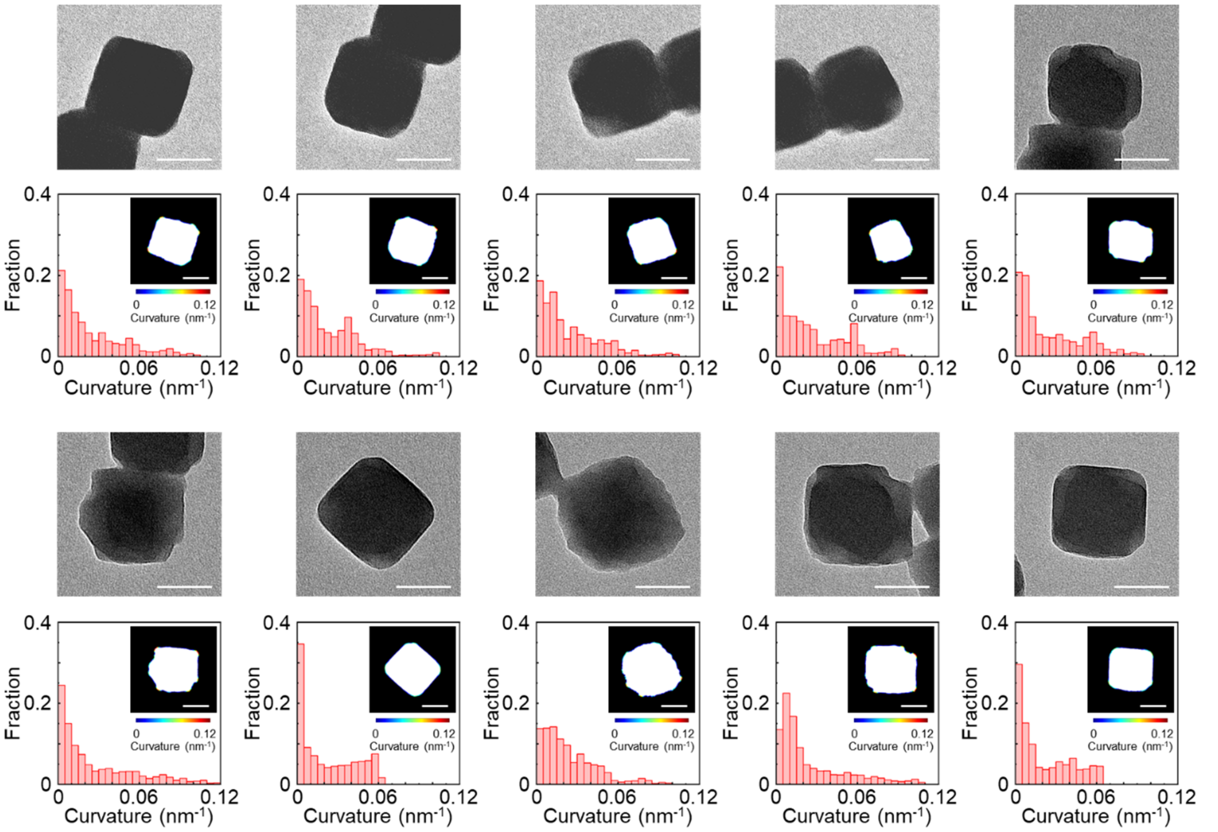

Figure S25. TEM images and curvature distribution of ten SIF_{imi} particles. Scale bar: 50 nm.

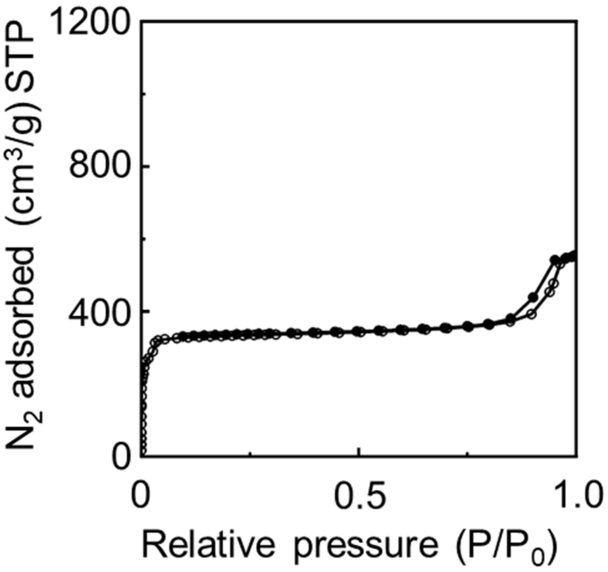

Figure S26. N_2 physisorption isotherms at 77 K for ZIF-8, illustrating the adsorption characteristics used to determine the BET specific surface area (991 m^2/g).

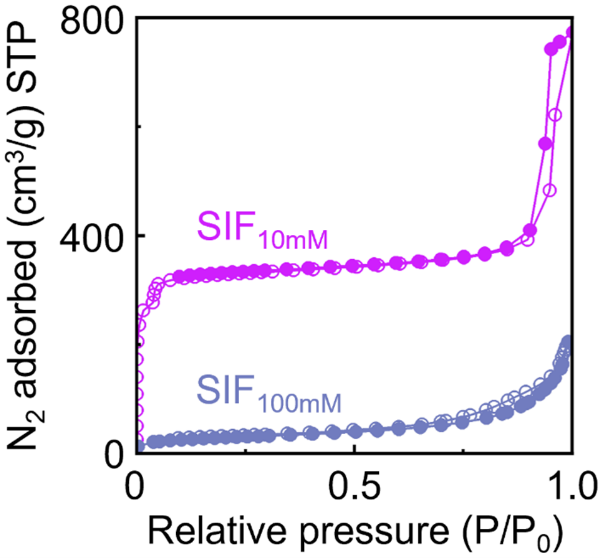

Figure S27. N_2 physisorption isotherms at 77 K for SIF_{10mM} (magenta) and SIF_{100mM} (light navy), illustrating the adsorption characteristics used to determine the BET specific surface area (987 m^2/g for SIF_{10mM} and 100 m^2/g for SIF_{100mM}).

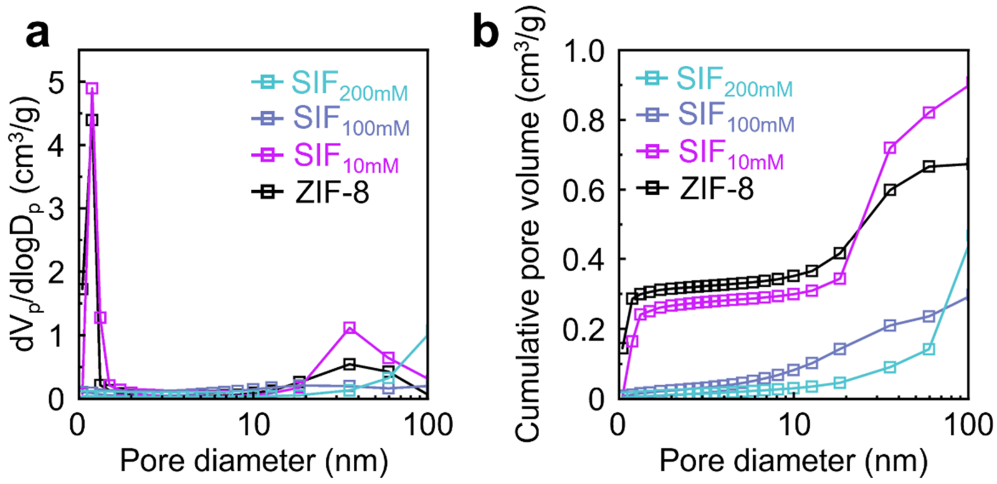

Figure S28. BJH pore size analysis of ZIF-8 (black), SIF_{10mM} (magenta), SIF_{100mM} (light navy), and SIF_{200mM} (light blue). (a) Differential pore volume distributions (dV_p/dlog d_p) plotted against pore diameter (d_p). (b) Cumulative pore volume curves as a function of pore diameter.

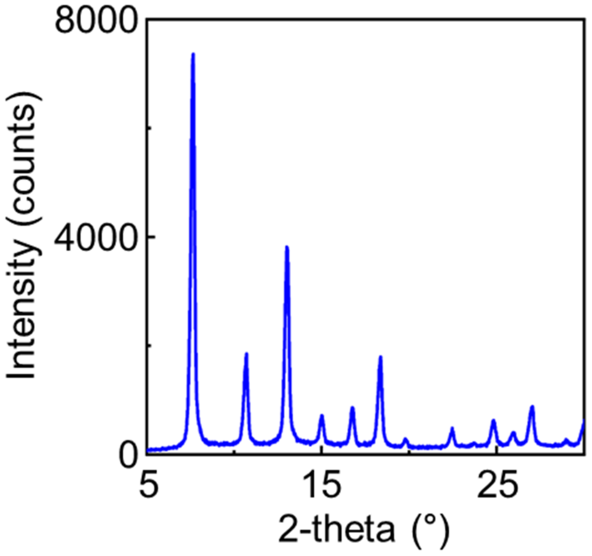

Figure S29. XRD pattern of SIF_{imi}.

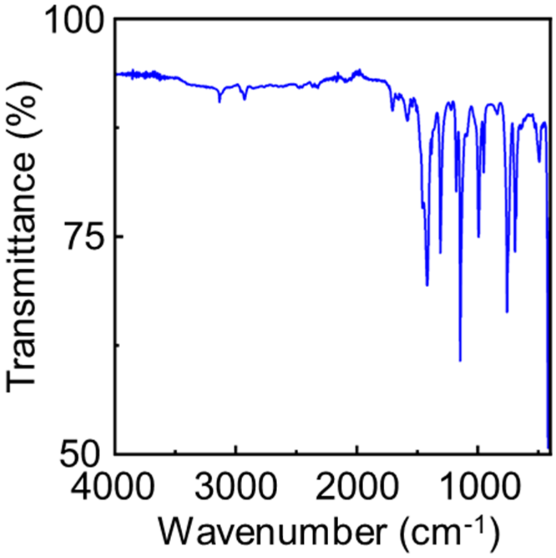

Figure S30. IR spectrum of SIF_{imi}.

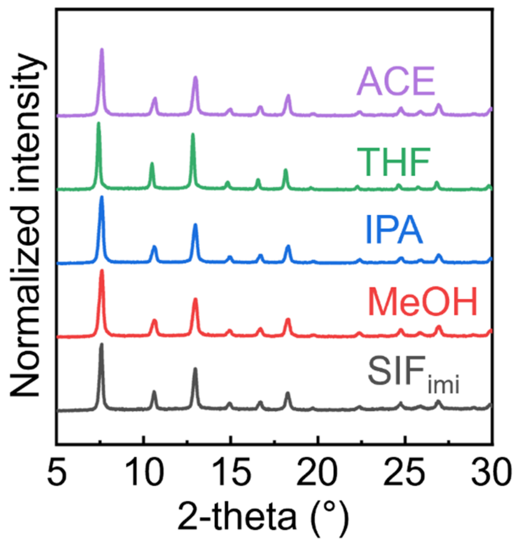

Figure S31. XRD patterns of SIF_{imi} (black) and after 48 h exposure to various solvents: MeOH (red), IPA (blue), THF (green), and ACE (purple). The retention of characteristic peaks after 48 h indicates that SIF_{imi} remains crystalline and stable in all tested solvents.

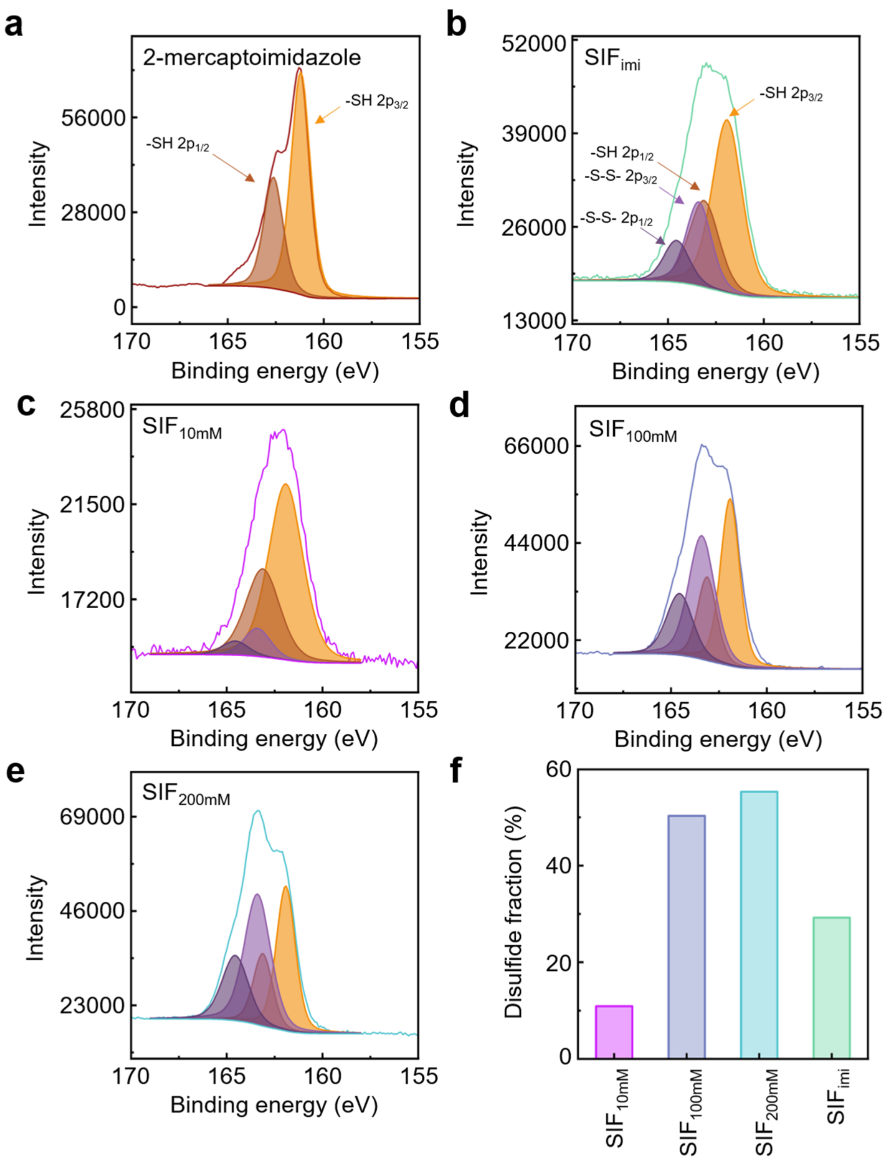

Figure S32. S 2p XPS spectra of (a) 2-mercaptoimidazole, (b) SIF_{imi}, (c) SIF_{10mM}, (d) SIF_{100mM}, and (e) SIF_{200mM}. In 2-mercaptoimidazole, the R-SH component appears at binding energies of 161.2 eV (S $2p_{3/2}$) and 162.63 eV (S $2p_{1/2}$). In SIF_{10mM}, SIF_{100mM}, SIF_{200mM}, and SIF_{imi}, the R-SH peaks shift slightly to 161.2 eV (S $2p_{3/2}$) and 163.12 eV (S $2p_{1/2}$). The disulfide (R-S-S-R) components are observed at 163.4 eV (S $2p_{3/2}$) and 164.56 eV (S $2p_{1/2}$). Each spectrum is overlaid with fitted component peaks (solid colored curves) and the overall experimental envelope (thin outline), highlighting the relative contributions of thiol and disulfide species in each sample. (f) Disulfide fraction (%) in SIF_{10mM}, SIF_{100mM}, SIF_{200mM}, and SIF_{imi}. The values were calculated from the deconvoluted S 2p XPS peak areas (disulfide vs. total sulfur, included by disulfide and thiol).

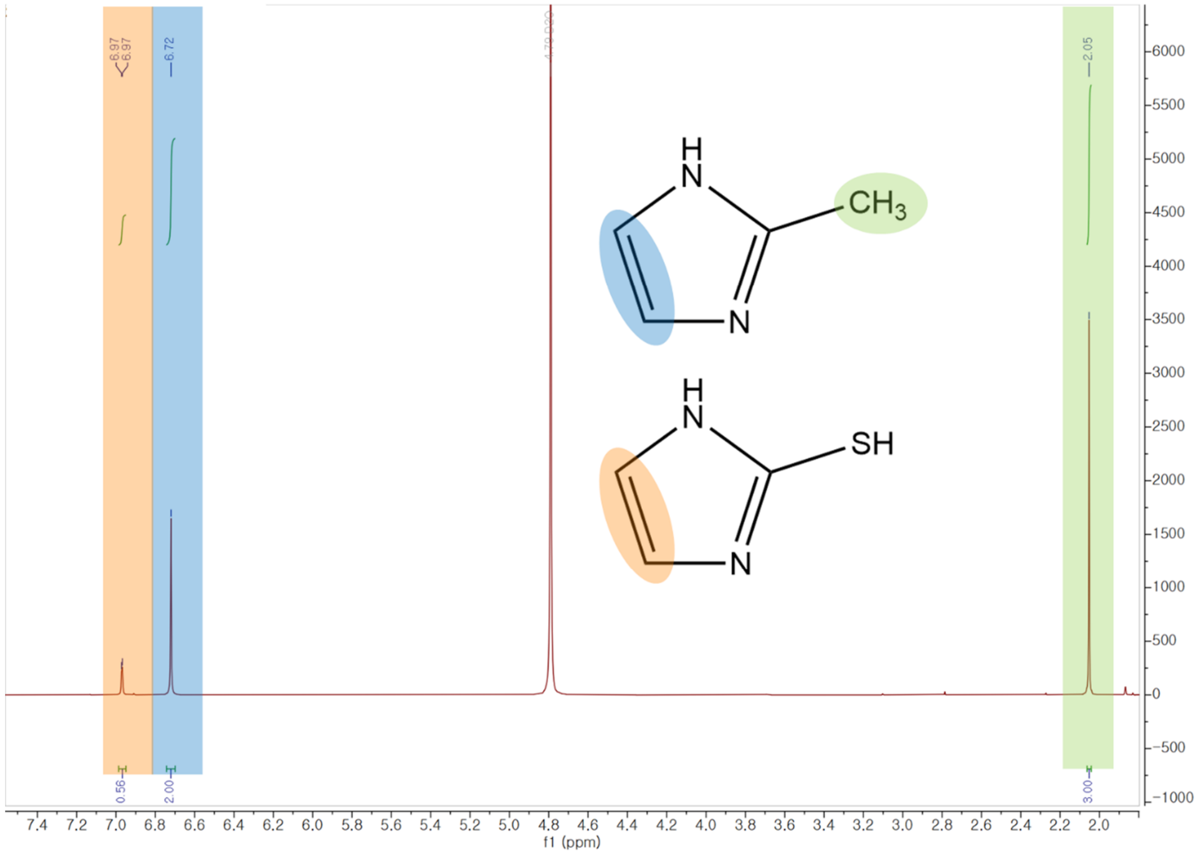

Figure S33. ^{1}H NMR spectrum of digested SIF_{imi}. The light orange-highlighted peak at 7.0 ppm corresponds to the two protons in the mercaptoimidazole ring (–CH=). The blue-highlighted peak at 6.7 ppm corresponds to the two protons in the imidazole ring (–CH=), while the green-highlighted peak at 2.0 ppm corresponds to the three protons in the methyl group ($–CH_3$).

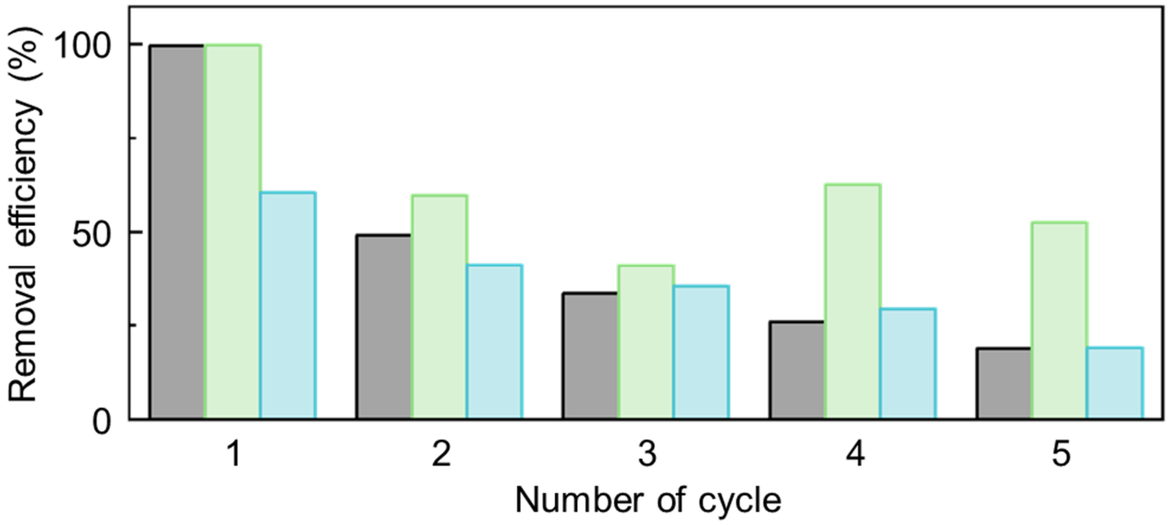

Figure S34. Hg^{2+} removal efficiency over five adsorption-desorption cycles by ZIF-8 (gray bars), SIF_{imi} (light green bars), and SIF_{200mM} (light blue bars) at an initial Hg^{2+} concentration of 7.2 mM (1444 ppm).

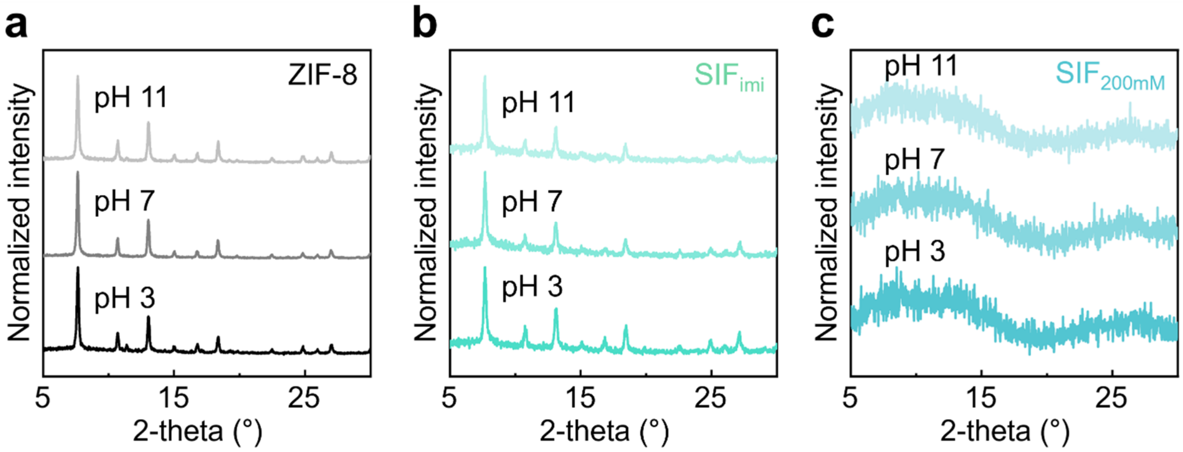

Figure S35. XRD patterns of (a) ZIF-8, (b) SIF_{imi}, and (c) SIF_{200mM} after adsorption of 10 ppm Hg^{2+} at different pH conditions. ZIF-8 at pH 3 exhibits an additional signal at $2\theta = 11.4°$, consistent with the formation of a Hg-imidazole phase via metal cation exchange under strongly acidic conditions.

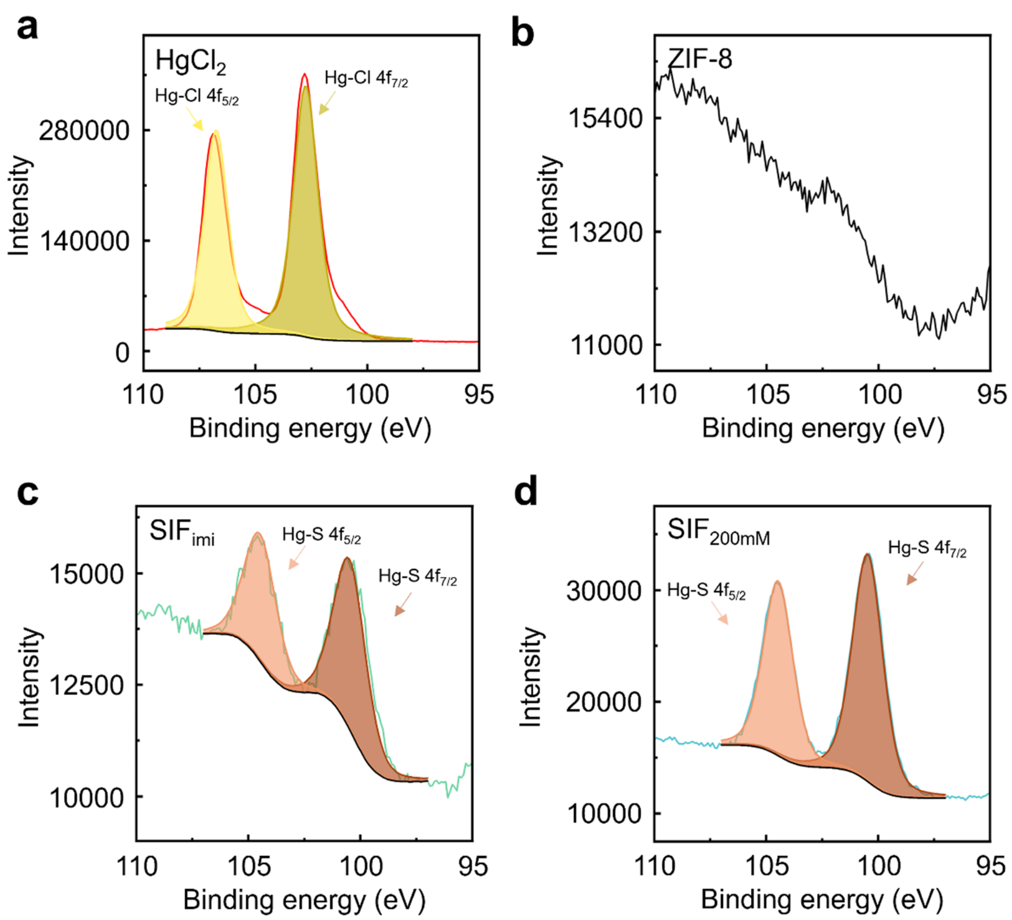

Figure S36. Hg 4f XPS spectra of (a) $HgCl_2$, (b) ZIF-8, (c) SIF_{imi}, and (d) SIF_{200mM} after uptake of 10 ppm Hg^{2+}. Hg-Cl appears at a higher binding energy than Hg-S.

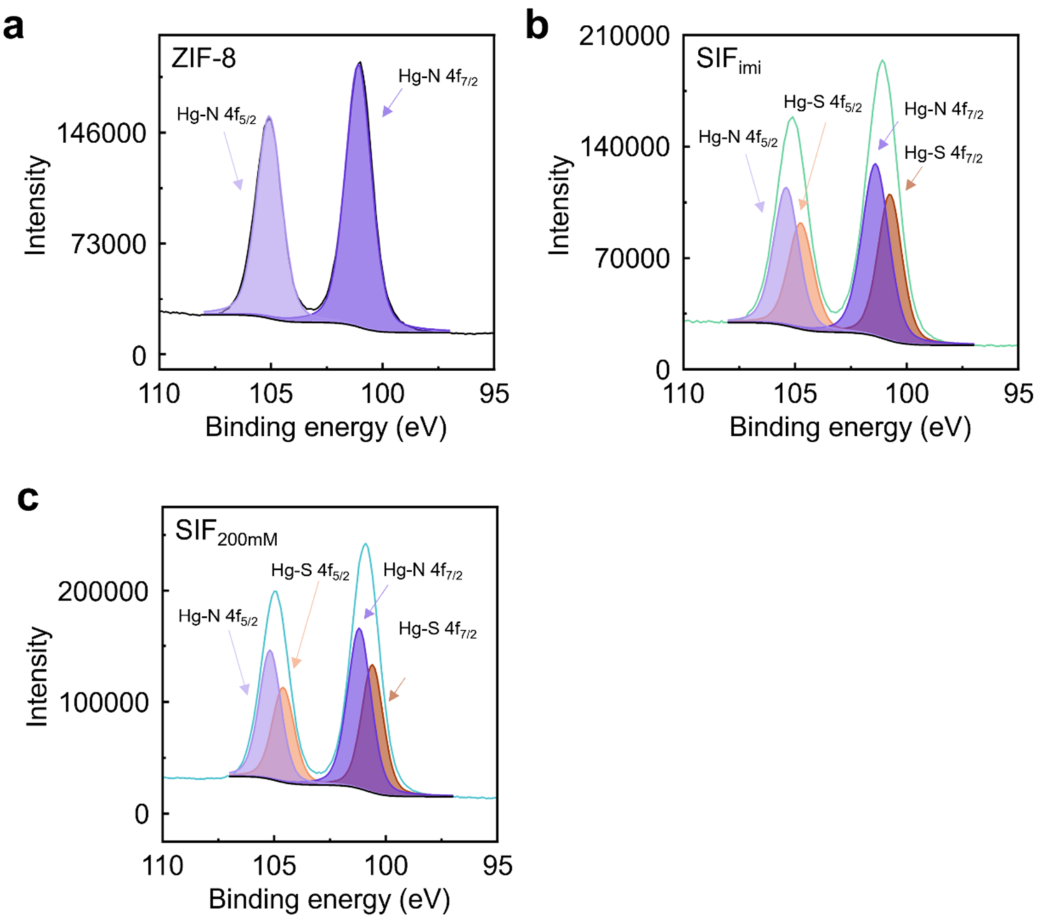

Figure S37. Hg 4f XPS spectra of (a) ZIF-8, (b) SIF_{imi}, and (c) SIF_{200mM} after uptake of 2005 ppm Hg^{2+}. Hg-N appears at a higher binding energy than Hg-S.

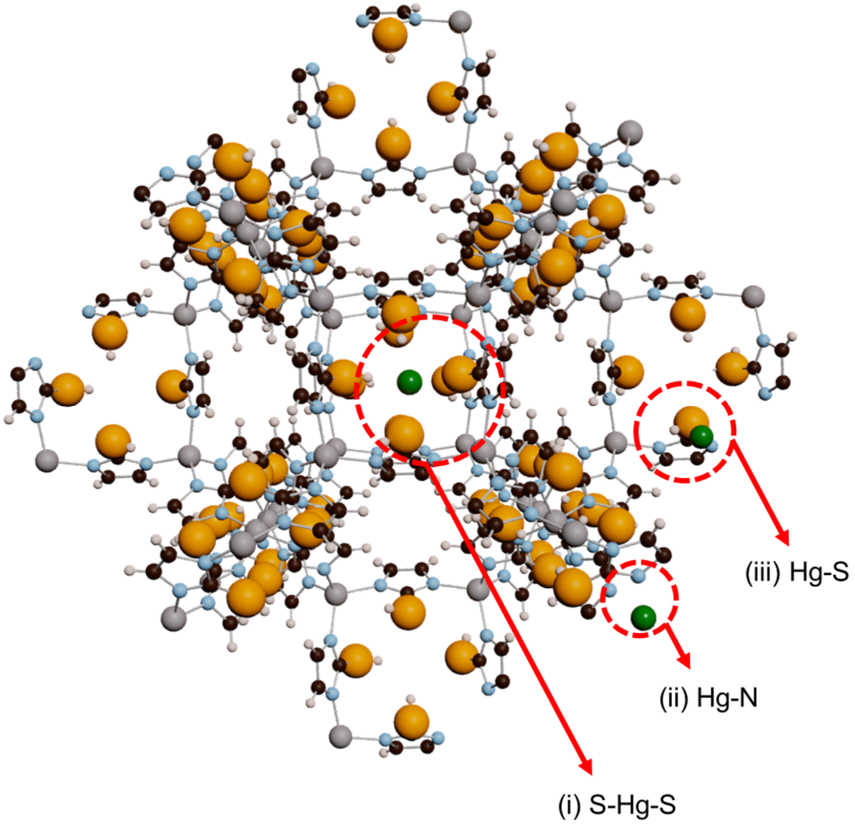

Figure S38. Three possible Hg^{2+} binding modes in SIF_{imi}. Red dashed circles indicate (i) window-site binding at dense sulfur clusters, (ii) metal-cation exchanged imidazole nitrogen sites, and (iii) single binding at exposed sparse thiol termini. Pale orange spheres represent sulfur atoms, green spheres represent Hg^{2+} ions, and grey sticks denote the framework.

Table S1. ^{1}H NMR peak integration values and calculated ligand exchange ratios of ZIF-8, SIF_{10mM}, SIF_{100mM}, SIF_{200mM}, SIF_{THF}, SIF_{ACE}, SIF_{IPA}, SIF_{EtOH}, SIF_{MeOH}, and SIF_{imi}.

Sample	2-mercaptoimidazole	2-methylimidazole		Ligand exchange ratio (%)
	sp^2 proton	sp^2 proton	sp^3 proton	
ZIF-8 (Figure S8)	0	1.96	3.00	0
SIF_{10mM} (Figure S9)	0	2.00	3.05	0
SIF_{100mM} (Figure S10)	3.65	2.00	3.22	64.6
SIF_{200mM} (Figure S11)	2	0.00	0.00	100.0
SIF_{THF} (Figure S15)	0.34	2.00	3.01	14.5
SIF_{ACE} (Figure S16)	0.51	2.00	3.06	20.3
SIF_{IPA} (Figure S17)	0.46	2.00	3.00	18.7
SIF_{EtOH} (Figure S18)	2.12	2.00	3.01	51.5
SIF_{MeOH} (Figure S19)	1.43	2.00	3.13	41.7
SIF_{imi} (Figure S33)	0.56	2.00	3.00	21.9

Table S2. BET specific surface area for ZIF-8, SIF_{10mM}, SIF_{100mM}, SIF_{200mM}, and SIF_{imi}.

Sample	BET specific surface area (m^2/g)
ZIF-8	991
SIF_{10mM}	987
SIF_{100mM}	100
SIF_{200mM}	52
SIF_{imi}	1013

Table S3. A list of Hg^{2+} ion adsorption capacities, recyclability, precursor costs, and selectivity for various MOFs and their composites. For simplicity, the costs of both metal and ligand precursors are provided. All materials show the highest selectivity for Hg^{2+} ions under competitive conditions with other metals ions when applied.

Sample	Adsorption capacity (mg/g)	Recycle number	Metal precursor	Price (USD/g)	Ligand precursor	Reagent manufacturer	Price (USD/g)	Other metal ions	Reference
SIF_{imi}	1504	5	$Zn(NO_3)_2{\cdot}6H_2O$	0.34	2-Methylimidazole	Sigma-Aldrich	0.63	Pb, Ni, Co, Fe, Cr, Cu	This work
MOF-808-SH	978	7	$ZrOCl_2{\cdot}8H_2O$	1.83	Fumaric acid		0.05	-	[4]
Zr-MSA	955	25	$ZrCl_4$	6.55	Mercaptosuccinic acid		0.69	Na, K, Ca, Mg, Pb, Cu, Cd, Ni, Zn	[5]
ZnS-ZIF-8	926	-	$Zn(NO_3)_2{\cdot}6H_2O$	0.34	2-Methylimidazole		0.63	Na, K, Fe, Zn, Mg, Ba, Mn, Ni, Ca, Al, Cu	[6]
$\{Ca^{II}Cu^{II}_6[(S,S)\text{-methox}]_3(OH)_2(H_2O)\}{\cdot}16H_2O$	900	-	$CuCl_2$	0.46	*L*-methionine methyl ester hydrochloride		7.96	-	[7]
$UiO\text{-}66\text{-}NH_2\text{-}(MAA)_2$	890	5	$ZrOCl_2{\cdot}8H_2O$	1.83	2-Aminoterephthalic acid		5.65	Na, K, Mg, Ca, Ni, Cu, Cd, Pb, Ag	[8]
UiO-66-SH	785	7	$ZrCl_4$	6.55	Terephthalic acid		0.51	Cd, Mn, Cu, Co, Ba, Zn, Ni, Pb	[9]
Zr-MSA-MA-1	715	4	$ZrCl_4$	6.55	Mercaptosuccinic acid		0.69	-	[10]
SH-HKUST-1	714	-	$Cu(NO_3)_2{\cdot}3H_2O$	0.91	Trimesic acid		1.38	-	[11]
UiO-66-DMTD	671	10	$ZrCl_4$	6.55	2,5-Diamino terephthalic acid	ChemScene LLC	143.19	Zn, Co, Ni, Cd, Mg, Fe, Ca, Cu	[12]
NENU-401	597	4	$Co(NO_3)_2{\cdot}6H_2O$	15.34	3,4-Dimethylthieno[2,3-b]thiophene-2,5-dicarboxylic acid		96	Pb, Fe, Zn, Na, K, Ca	[13]
In_2S_3@MIL-101	518	3	$Cr(NO_3)_3{\cdot}9H_2O$	0.43	Terephthalic acid	Sigma-Aldrich	0.51	-	[14]
SNN-MIL-125(Ti)@Fe_3O_4	511	4	$Ti[OCH(CH_3)_2]_4$	0.24	2-Aminoterephthalic acid		5.65	-	[15]
SH-MIL-68(In)	450	5	$In(NO_3)_3$	9.94	2-Aminoterephthalic acid		5.65	Cd, Co, Mg, Cu, Mn, Ni, Pb, Zn	[16]
Zr-DMBD	427	3	$ZrOCl_2{\cdot}8H_2O$	1.83	2,5-Dimercaptoterephthalic acid		871.58	Ca, Cd, Co, Cu, Hg, Mg, Ni, Pb	[17]
ZrOMTP	403	3	$ZrCl_4$	6.55	4-Bromo-2,6-difluorobenzoic acid		13.7	-	[18]
DUT-67-SH-3	372	4	$ZrOCl_2{\cdot}8H_2O$	1.83	2,5-Thiophenedicarboxylic acid		6.2	-	[19]
Zr-L4	322	2	$ZrCl_4$	6.55	1,4-Dibromo-2,5-difluorobenzene	ChemScene LLC	0.34	-	[20]

MFC-S	282	5	$ZrCl_4$	6.55	Terephthalic acid		0.51	Cd, Co, Cr, Cu, Mn, Ni, Pb, Zn	[21]
Zr-M1	275	4	$ZrCl_4$	6.55	Methyl 3-hydroxybenzoate		1.73	-	[22]
Fe_3O_4@SiO_2@HKUST-1/Bi-I	264	-	$CuCl_2{\cdot}H_2O$	0.74	Trimesic acid		1.38	Hg, Cd, Co, Cr, Mn, Ni, Pb, Zn	[23]
MIL-101-SH	250	5	$Cr(NO_3)_3{\cdot}9H_2O$	0.43	Terephthalic acid		0.51	-	[24]
UiO66-BAT-SH	243	5	$ZrCl_4$	6.55	2,5-Dihydroxyterephthalic acid		71.63	Mg, Zn, Ca, Ni, Pd, Al, Cu	[25]
UiO-66-$(SH)_2$	236	3	$ZrOCl_2{\cdot}8H_2O$	1.83	2,5-Dimercaptoterephthalic acid	Sigma-Aldrich	871.58	Fe, Cu	[26]
SH@SiO_2/$Cu_3(BTC)_2$	210	15	$Cu(OAc)_2{\cdot}H_2O$	0.91	Trimesic acid		1.38	-	[27]
Zr-DMBD	172	3	$ZrCl_4$	6.55	2,5-Dimercapto-1,4-benzenedicarboxylic acid		871.58	Mg, Ca, Pb, Cd, Zn, Cu, Ni, Co	[28]
$[Cu_4O(BDC)]_n$-SH	170	3	$Cu(NO_3)_2{\cdot}3H_2O$	0.91	Terephthalic acid		0.51	Na, K, Ca, Mg, Fe, Zn	[29]
ZIF-67@GO	131	5	$Co(NO_3)_2.6H_2O$	15.34	2-Methylimidazole		0.63	-	[30]
UiO-66-SH	110	5	$ZrCl_4$	6.55	Terephthalic acid		0.51	-	[24]
ZIF-90-HS	22	-	$Zn(NO_3)_2 \cdot 6H_2O$	0.34	Imidazolate-2-carboxyaldehyde		83.42	-	[31]

Table S4. XPS Hg 4f binding energies, peak areas, and area ratios for $HgCl_2$, Hg^{2+}-adsorbed ZIF-8, SIF_{imi}, and SIF_{200mM} at different Hg^{2+} adsorption concentrations.

Hg^{2+} ion (ppm)	Sample	Binding	Hg $4f_{7/2}$ B.E.	Hg $4f_{7/2}$ area	Hg $4f_{5/2}$ B.E.	Hg $4f_{5/2}$ area	Hg $4f_{7/2}$ area ratio	Hg $4f_{5/2}$ area ratio	ΔB.E.
Control	$HgCl_2$	Hg-Cl	102.75	543681	106.75	407761	4.0	3.0	4.0
10	SIF_{imi}	Hg-S	100.47	7871	104.47	5903	4.0	3.0	4.0
	SIF_{200mM}	Hg-S	100.47	39467	104.47	29600	4.0	3.0	4.0
2005	ZIF-8	Hg-N	101.08	290810	105.08	218107	4.0	3.0	4.0
	SIF_{imi}	Hg-N	101.40	189245	105.40	141934	4.0	3.0	4.0
		Hg-S	100.75	143735	104.75	107801	4.0	3.0	4.0
	SIF_{200mM}	Hg-N	101.19	217012	105.19	162759	4.0	3.0	4.0
		Hg-S	100.60	160723	104.60	120542	4.0	3.0	4.0

References

[1] S. Kim, S. Woo, C. Bae, Y. Jeon, Y. Seong, S. Li, J. Kim, *Inorg. Chem. Front.* **2024**, *11*, 2608.

[2] J. Lee, C. Bae, Z. Ou, S. Park, J. Kim, J. Kim, *Nanoscale Adv.* **2021**, *3*, 1927.

[3] K. Pillay, E. M. Cukrowska, N. J. Coville, *Microchem. J.* **2013**, *108*, 124.

[4] C. Ji, Y. Ren, H. Yu, M. Hua, L. Lv, W. Zhang, *Chem. Eng. J.* **2022**, *430*, 132960.

[5] K. Fu, X. Liu, C. Lv, J. Luo, M. Sun, S. Luo, J. C. Crittenden, *Environ. Sci. Technol.* **2022**, *56*, 2677.

[6] F. Liu, W. Xiong, X. Feng, L. Shi, D. Chen, Y. Zhang, *J. Hazard. Mater.* **2019**, *367*, 381.

[7] M. Mon, F. Lloret, J. Ferrando-Soria, C. Martí-Gastaldo, D. Armentano, E. Pardo, *Angew. Chem. Int. Ed.* **2016**, *55*, 11167.

[8] I. Abdulazeez, H. A. Asmaly, O. C. S. Al-Hamouz, N. Baig, K. M. AlAqad, M. A. Raji, K. H. Ahmed, *New J. Chem.* **2025**, *49*, 3096.

[9] J. Li, Y. Liu, Y. Ai, A. Alsaedi, T. Hayat, X. Wang, *Chem. Eng. J.* **2018**, *354*, 790.

[10] X. Gao, B. Liu, X. Zhao, *Chemosphere* **2023**, *317*, 137891.

[11] F. Ke, L.-G. Qiu, Y.-P. Yuan, F.-M. Peng, X. Jiang, A.-J. Xie, Y.-H. Shen, J.-F. Zhu, *J. Hazard. Mater.* **2011**, *196*, 36.

[12] L. Fu, S. Wang, G. Lin, L. Zhang, Q. Liu, J. Fang, C. Wei, G. Liu, *J. Hazard. Mater.* **2019**, *368*, 42.

[13] S.-Y. Jiang, W.-W. He, S.-L. Li, Z.-M. Su, Y.-Q. Lan, *Inorg. Chem.* **2018**, *57*, 6118.

[14] L. Liang, L. Liu, F. Jiang, C. Liu, D. Yuan, Q. Chen, D. Wu, H.-L. Jiang, M. Hong, *Inorg. Chem.* **2018**, *57*, 4891.

[15] J. Li, G. Lin, F. Tan, L. Fu, B. Zeng, S. Wang, T. Hu, L. Zhang, *J. Colloid Interface Sci.* **2023**, *651*, 659.

[16] G.-P. Li, K. Zhang, P.-F. Zhang, W.-N. Liu, W.-Q. Tong, L. Hou, Y.-Y. Wang, *Inorg. Chem.* **2019**, *58*, 3409.

[17] X. Yan, J. Feng, P. Li, J. Li, B. Ren, S. Gao, R. Cao, *Inorg. Chem. Commun.* **2021**, *131*, 108796.

[18] M.-Q. Li, Y.-L. Wong, T.-S. Lum, K. Sze-Yin Leung, P. K. S. Lam, Z. Xu, *J. Mater. Chem. A* **2018**, *6*, 14566.

[19] X. Zhao, X. Gao, Y.-N. Zhang, M. Wang, X. Gao, B. Liu, *J. Colloid Interface Sci.* **2023**, *631*, 191.

[20] Y. He, Y.-L. Hou, Y.-L. Wong, R. Xiao, M.-Q. Li, Z. Hao, J. Huang, L. Wang, M. Zeller, J. He, Z. Xu, *J. Mater. Chem. A* **2018**, *6*, 1648.

[21] L. Huang, M. He, B. Chen, B. Hu, *J. Mater. Chem. A* **2016**, *4*, 5159.

[22] Y.-L. Hou, K.-K. Yee, Y.-L. Wong, M. Zha, J. He, M. Zeller, A. D. Hunter, K. Yang, Z. Xu, *J. Am. Chem. Soc.* **2016**, *138*, 14852.

[23] L. Huang, M. He, B. Chen, B. Hu, *J. Mater. Chem. A* **2015**, *3*, 11587.

[24] F. Liu, Xiong ,Wenjing, Feng ,Xinrui, Cheng ,Ge, Shi ,Lei, Chen ,Dawei, Y. and Zhang, *Environ. Technol.* **2020**, *41*, 3094.

[25] X. Huang, M. Zhao, M. Xu, J. Hu, J. Wang, X. Miao, D. Xie, *J. Mol. Struct.* **2024**, *1308*, 137972.

[26] K. Leus, J. P. H. Perez, K. Folens, M. Meledina, G. Van Tendeloo, G. Du Laing, P. Van Der Voort, *Faraday Discuss.* **2017**, *201*, 145.

[27] M. R. Sohrabi, *Microchim. Acta* **2014**, *181*, 435.

[28] L. Ding, X. Luo, P. Shao, J. Yang, D. Sun, *ACS Sustainable Chem. Eng.* **2018**, *6*, 8494.

[29] Y. Wu, G. Xu, W. Liu, J. Yang, F. Wei, L. Li, W. Zhang, Q. Hu, *Microporous Mesoporous Mater.* **2015**, *210*, 110.

[30] A. M. Fallatah, H. U. R. Shah, K. Ahmad, M. Ashfaq, A. Rauf, M. Muneer, M. M. Ibrahim, Z. M. El-Bahy, A. Shahzad, A. Babras, *Heliyon* **2022**, *8*, e10936.

[31] S. Bhattacharjee, Y.-R. Lee, W.-S. Ahn, *CrystEngComm* **2015**, *17*, 2575.
